# Supplementary material for: Resonant tunneling driven metal-insulator transition in double quantum-well structures of strongly correlated oxide
Source: Nat Commun. 2021 Dec 3;12:7070. doi: 10.1038/s41467-021-27327-z (PMC8642393; doi:10.1038/s41467-021-27327-z)
Supplement: Supplementary file 1 — Supplementary Information [file 41467_2021_27327_MOESM1_ESM.pdf]

Supplementary Information

Resonant tunneling driven metal-insulator transition in double quantum-well structures of strongly correlated oxide

R. Yukawa[1,†], M. Kobayashi[1,†], T. Kanda[2,†], D. Shiga[1,2,†], K. Yoshimatsu[2], S. Ishibashi[3], M. Minohara[1], M. Kitamura[1], K. Horiba[1], A. F. Santander-Syro[4], and H. Kumigashira[1,2,*]

[1] *Photon Factory, Institute of Materials Structure Science, High Energy Accelerator Research Organization (KEK), Tsukuba, 305–0801, Japan*

[2] *Institute of Multidisciplinary Research for Advanced Materials (IMRAM), Tohoku University, Sendai, 980–8577, Japan*

[3] *Research Center for Computational Design of Advanced Functional Materials, National Institute of Advanced Industrial Science and Technology (AIST), Tsukuba, Ibaraki 305-8568, Japan*

[4] *Université Paris-Saclay, CNRS, Institut des Sciences Moléculaires d'Orsay, 91405, Orsay, France*

[†] These authors contributed equally to this work.

[*] Correspondence and requests for materials should be addressed to H.K. (e-mail: kumigashira@tohoku.ac.jp).

Supplementary Note 1. Definition of "resonant tunneling"

In general, resonant tunneling is a phenomenon that an electron passes through the barrier layers in quantum well (QW) structures without energy decay; it occurs when the energy of an incoming electron matches that of an electron confined in the two potential barriers[1]. Although one of the two barriers is a vacuum for the top QW structures in the present double QW structures, the metal-insulator transition occurs through the significant spread of the wavefunction owing to the energy matching between the QW states (n = 2 in the bottom QW structure and n = 1 in the top QW structure). Hence, we use the term "resonant tunneling".

Supplementary Note 2. Why *W* is assumed to be unchanged

According to the Mott-Hubbard theory[2], the bandwidth (W) is defined as the kinetic energy of an electron moving between sites, whereas the Coulomb interaction (U) is defined as the on-site Coulomb repulsion between electrons. In the QW structures considered here, W is primarily determined by the spread of the wavefunction in the plane, because the spread of the wavefunction perpendicular to the plane is considerably smaller than that in the plane, owing to the quantum confinements. Therefore, the spillage of the wavefunction along the direction perpendicular to the plane has a negligible influence on the W value. Meanwhile, the effective U is determined by the Coulomb integral; hence, the spillage of the wavefunction largely affects U.

Supplementary Note 3. Structure plot of d_{zx} states for $SrVO_3$ QW structures for designing resonant tunneling in double QW structures

To design double quantum well (QW) structures for resonant tunneling (RT), we utilized the structure plot (the plot of quantization energies as a function of $SrVO_3$ layer thickness) of d_{zx} states for $SrVO_3$ QW structures, as shown in Supplementary Fig. 1. Results with quantum numbers n = 1–4 are shown in red, orange, green, and blue, respectively[3–6]. The solid lines are predictions for the QW states from the tight-binding (TB) calculation in the renormalized scheme[7]. The TB results reproduce the experimental results, although there are discrepancies between the experiment and the calculation. The discrepancies seem to become larger by approaching the quantization energies to the Fermi level (E_F), probably reflecting the unusual band renormalization of the subbands near E_F (Supplementary Refs. 3–6).

By extrapolating the data, the original quantized energy of n = 1 states for a 2-ML $SrVO_3$ QW structure is expected to be in the range of 200–300 meV (hatched green region), although the 2-ML $SrVO_3$ QW is a Mott insulator and its original QW state is localized as the lower Hubbard band (see Fig. 2 in the main text and Supplementary Fig. 18). Judging from the structure plot, we concluded that a 6-ML $SrVO_3$ QW structure is an optimal counterpart in the double QW structure to induce the RT effect between two energetically close QW states: the quantization energy of n = 2 states for the 6-ML $SrVO_3$ QW structure is close to the original quantized energy of n = 1 states for a 2-ML $SrVO_3$ QW structure. The existence of the energetically close QW states in both the top and bottom QW structures suggests the hybridized nature of the envelope wavefunctions of the two subbands, leading to the RT effect between the two QWs. Based on the structure plot, we employed the $V_2T_LV_6$ double QW structures in the present study.

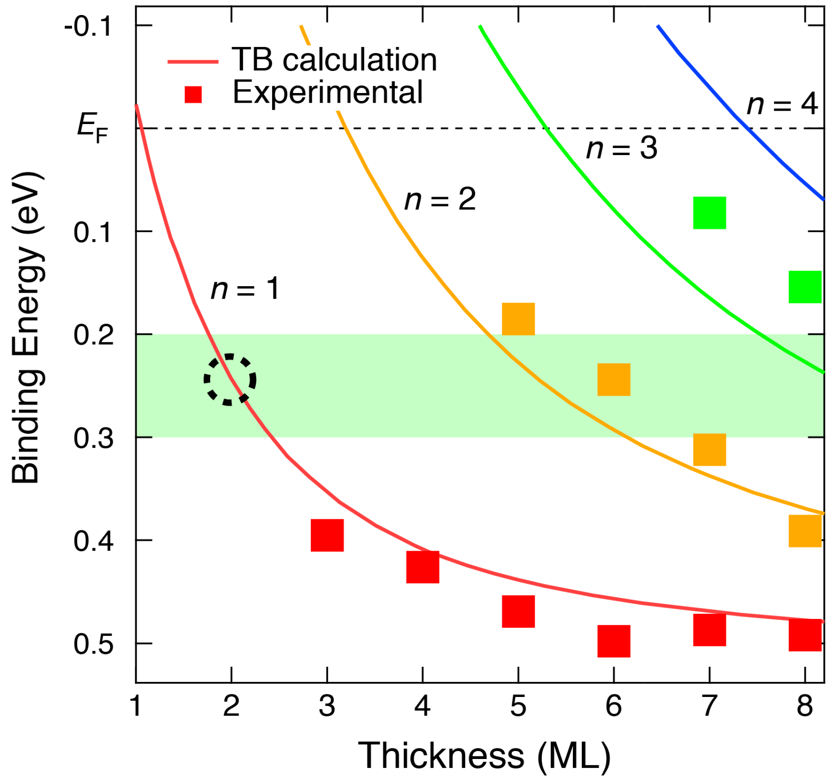

Supplementary Figure 1: Structure plot of d_{zx} states for $SrVO_3$ QW structures. Structure plots showing the energy positions of the respective QW states of d_{zx} states (data markers) versus the layer thickness of $SrVO_3$. The data are taken from Supplementary Refs. 3–6. The solid lines are the predictions for the QW states, obtained from the TB calculation in the renormalized scheme[7]. Note that the TB results are in good agreement with the results of DFT calculations with a normalization factor $Z = 0.55$ (Supplementary Ref. 7). A dotted-line circle indicates the expected original quantized energy of $n = 1$ states for a 2-ML $SrVO_3$ QW structure.

Supplementary Note 4. Sample growth and characterization

The double quantum well (QW) structures of (N-ML $SrVO_3$)/(L-ML $SrTiO_3$)/(M-ML $SrVO_3$) ($V_NT_LV_M$) were fabricated onto atomically flat TiO_2-terminated 0.05 wt% Nb-doped $SrTiO_3$ (Nb:STO) (001) substrates in a laser molecular-beam epitaxy chamber that is connected under ultrahigh vacuum to an angle-resolved photoemission spectroscopy (ARPES) system at BL-2A MUSASHI of Photon Factory, KEK (see Supplementary Note 6). Sintered $SrVO_y$ and $SrTiO_3$ pellets were used as ablation targets. An Nd-doped yttrium aluminum garnet laser was used for target ablation in its frequency-tripled mode (λ = 355 nm) at a repetition rate of 1 Hz. During the deposition of both layers, the substrate temperature was maintained at 900°C, and the oxygen pressure was maintained at less than 10^{-8} Torr (Supplementary Refs. 3–6,8). Note that each layer of the double QW structures was grown under the same conditions as those of previously reported QW structures, wherein coherent growth on the substrate and the formation of a chemically abrupt $SrVO_3$/ $SrTiO_3$ interface were achieved[3–6,8]. During the growth of each layer, the thickness was precisely controlled at the atomic scale by monitoring the intensity oscillation of reflection high-energy electron diffraction (RHEED) spots. As a typical example, the RHEED intensity oscillations during the growth of a $V_6T_2V_2$ heterostructure are shown in Supplementary Fig. 2. The clear RHEED oscillations during the growth of the $SrVO_3$ and $SrTiO_3$ layers indicate a layer-by-layer growth. The period of oscillation corresponds to the deposition of one monolayer (ML) of $SrVO_3$ and $SrTiO_3$, which was also confirmed using the deposition rate estimated from grazing-incidence x-ray reflectivity measurements. The high surface quality, surface flatness, and epitaxial growth of each layer are confirmed by the RHEED patterns which show sharp streak patterns and Kikuchi lines at all growth stages. Furthermore, the almost identical RHEED pattern after $SrVO_3$-layer deposition indicates that the top and bottom $SrVO_3$ layers are grown with almost identical crystallinity.

The surface morphologies of the prepared QW structures were analyzed by atomic force microscopy (AFM). Atomically flat surfaces with step-and-terrace structures, which reflected the morphology of the Nb:STO substrate, were clearly observed for all samples, indicating that not only the surface but also the buried interfaces were atomically flat. As a typical example, the AFM image of a double QW structure after fabrication is presented in Supplementary Fig. 3. A clear step-and-terrace structure is observed even after fabricating the $V_NT_LV_M$ double QW structure. The surface structures and cleanness of the measured double QW structures were also

confirmed via low-energy electron diffraction (LEED) and core-level photoemission measurements, respectively. The LEED patterns exhibited sharp 1 × 1 spots with some superstructure spots of $\sqrt{2}\times\sqrt{2}$-R45° for all samples. The prepared films were transferred to the photoemission (PES) chamber under an ultrahigh vacuum of 10^{-10} Torr. The in-vacuum transfer was necessary to avoid degradation of the $SrVO_3$ surfaces upon exposure to air (Supplementary Fig. 11). The surface cleanness and stoichiometry of the samples were carefully characterized by analyzing the relative intensities of the relevant core levels.

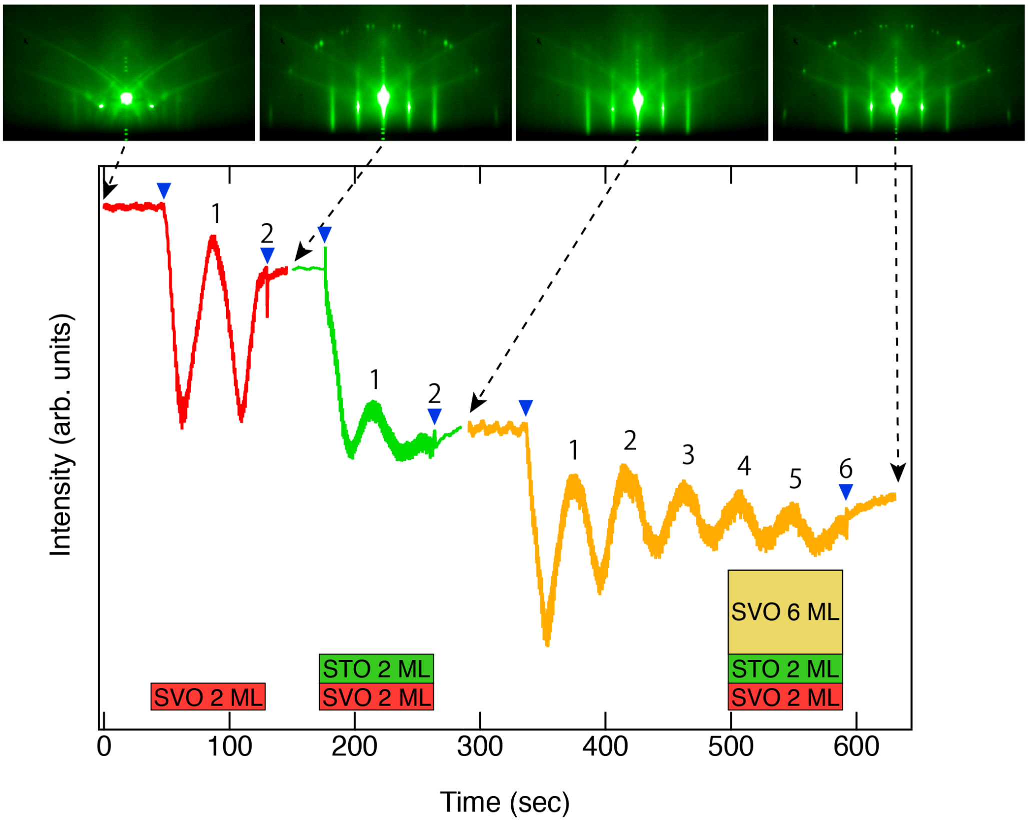

Supplementary Figure 2: RHEED intensity oscillations during the growth of the $V_6T_2V_2$ double quantum-well structures. The start and end of the deposition for each constituent layer are indicated by blue triangles. RHEED patterns taken at each growth stage in this fabrication procedure are shown on the top of the graph. The inset shows schematic side views of the structures at each fabrication stage.

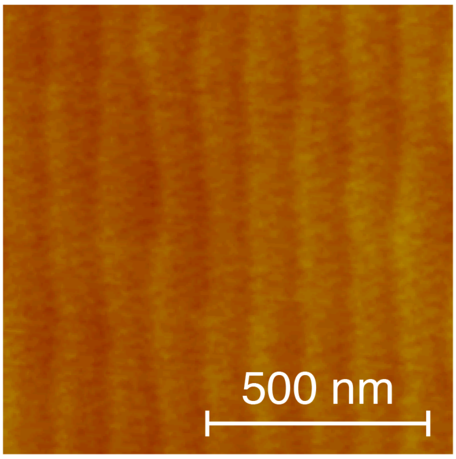

Supplementary Figure 3: Typical AFM image of $V_NT_LV_M$ quantum-well structures taken after growth. The scan area is 1 × 1 μm^2. An atomically flat surface with step-and-terrace structures, which reflects the morphology of the Nb:STO substrate, is clearly observed, indicating that not only the surface but also the buried interfaces are atomically flat.

Supplementary Note 5. Formation of a chemically abrupt interface

5.1 Core-level analysis for $SrVO_3/SrTiO_3$ interfaces

We present evidence that the prepared double QW structures have atomically and chemically abrupt $SrVO_3/SrTiO_3$ interfaces. This feature is a precondition to the present study on the double QW structures, and the evidence demonstrates that the precondition is fulfilled. Supplementary Figure 4 shows the Ti 2*p* core-level spectra of $SrVO_3/SrTiO_3$ with varying $SrVO_3$ overlayer thickness *t*, as well as a $SrTiO_3$ substrate as a reference. The spectra shown in Supplementary Fig. 4a are normalized to the incident photon flux, reflecting the attenuation of the Ti 2*p* signal from the buried $SrTiO_3$ by the $SrVO_3$ overlayers. The intensity of the Ti 2*p* core level I_{Ti} is steeply reduced with increasing *t* and almost disappears at $t = 3$ nm, suggesting the formation of a chemically abrupt interface. The shift of the energy position by 1.0 eV is attributed to the formation of a Schottky barrier at the heterointerface between metallic $SrVO_3$ and *n*-type oxide-semiconductor Nb:$SrTiO_3$ (Supplementary Refs. 3,4). In addition, the line shape of the Ti 2*p* core level maintains its original Ti^{4+} feature, indicative of the invariance of the chemical environments even at the interface, although slightly asymmetric spectral behavior is observed for thicker $SrVO_3$ films owing to the formation of the Schottky barrier[3,9].

To evaluate the length of the possible interdiffusion, we plot I_{Ti} as a function of *t* in Supplementary Fig. 4b, comparing it with the calculated photoemission attenuation function $I_{Ti} = e^{-t/\lambda}$, where λ is the inelastic mean-free path of photoelectrons and 1 ML of the $SrVO_3$ overlayer corresponds to 0.387 nm. The excellent agreement between the experiment and calculation indicates that the constituent cations are not intermixed with each other across the interface within the experimental margins. Since both oxides share a common A-site composition (SrO), the results indicate the formation of chemically abrupt interfaces. To highlight the chemical abruptness of the present films, we simulated the intensity attenuation by assuming the formation of an intermixing layer of 0.2–0.7 nm (Supplementary Ref. 10). The results are overlaid in the inset of Supplementary Fig. 4b. From the simulation results, the interdiffusion length of the present film is estimated to be less than 0.2 nm. Furthermore, the root-mean-square of surface roughness R_{RMS} of the films, as well as the substrate, was less than 0.2 nm irrespective of the film thickness (see Supplementary Fig. 3). These results indicate that the atomically and chemically abrupt interfaces, as well as the atomically flat surfaces, are achieved in the present films, as required for our spectroscopic measurements.

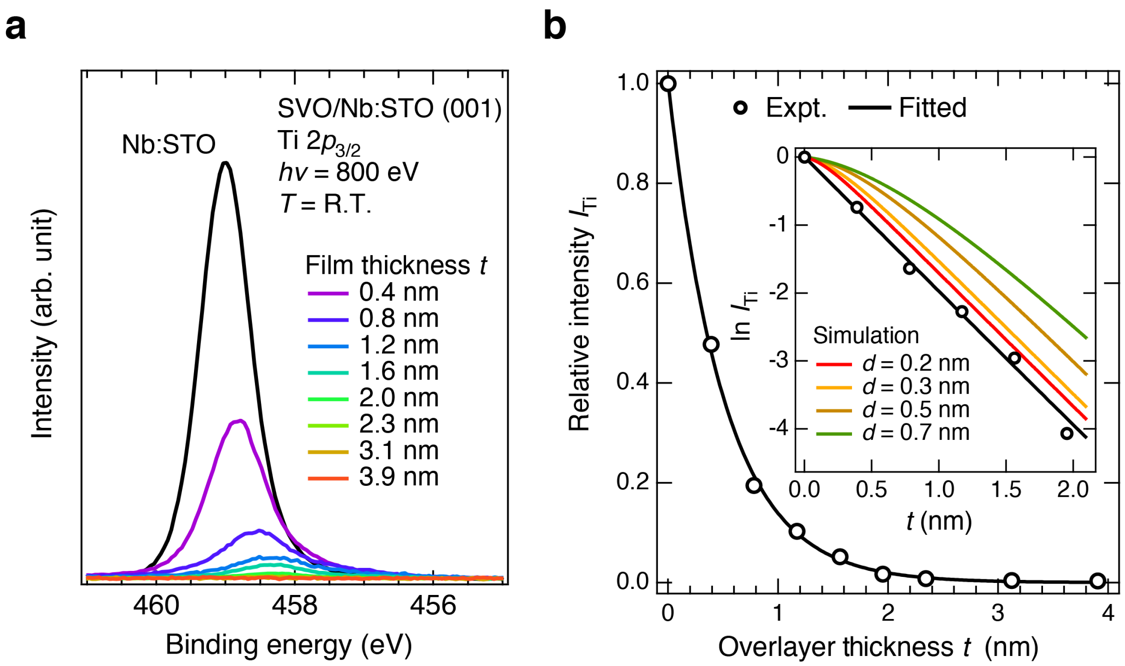

Supplementary Figure 4: Core-level analysis for $SrVO_3/SrTiO_3$ interfaces. **a**, Thickness dependence of Ti $2p_{3/2}$ core-level spectra measured at $h\nu = 800$ eV for $SrVO_3$/Nb:$SrTiO_3$(001) QW structures. Each spectrum is normalized to the incident photon flux; hence, the intensity reduction with increasing $SrVO_3$ overlayer thickness t reflects the attenuation of the Ti $2p$ signal from the buried $SrTiO_3$ by the overlayer. **b**, Plot of relative intensities of the background-subtracted Ti $2p_{3/2}$ core-level spectra I_{Ti} as a function of t. The experimental points are fitted to the photoemission attenuation function with $\lambda = 0.51(2)$ nm. Note that the excellent agreement between the experiment and calculation also indicates the successful digital control of overlayer thickness. The inset shows the logarithm plot of I_{Ti} with respect to t in comparison with the simulation curves that assume an intermixing of V and Ti ions at the interface with interdiffusion lengths d of 0.2–0.7 nm. From the comparison, it is clear that the interdiffusion length of the present film is less than 0.2 nm, which is approximately half of the c-axis length of ~0.39 nm.

5.2 Core-level analysis for digital control of $SrTiO_3$ layers

The excellent agreement between the experiment and calculation presented in Supplementary Fig. 4 also indicates the successful digital control of the $SrVO_3$ layer thickness. We also evaluate the thickness of a $SrTiO_3$ barrier layer sandwiched between $SrVO_3$ layers using core-level spectra.

Supplementary Figures 5a and 5b show the core-level spectra of 2-ML $SrVO_3$/ L-ML $SrTiO_3$/ 6-ML $SrVO_3$ ($V_2T_LV_6$) double QW structures with varying $SrTiO_3$ barrier layer thickness L. It should be noted that the spectra were recorded using the same samples as those employed for the ARPES measurements shown in Fig. 2 in the main text (Supplementary Figs. 15 and 19). With increasing L, the intensity of the Ti 2p core-level peak emitted from the $SrTiO_3$ barrier layers increases and almost saturates around L = 10 ML. These behaviors of the core-level intensity with respect to L strongly suggest the successful digital control of $SrTiO_3$ barrier layers, as well as the formation of chemically abrupt interfaces between $SrVO_3$ and $SrTiO_3$.

To quantitatively evaluate the accuracy of the digital thickness control, we plot the core-level intensity of the Ti 2p core level (I_{Ti}) in Supplementary Fig. 6 as a function of L, and we compare the results with the simulation based on the photoelectron attenuation function. Assuming a chemically abrupt interface, I_{Ti} is given by the following equation:

$$I_{\mathrm{Ti}} \propto \exp\left[-\frac{(L+6)a}{\lambda}\right] - \exp\left(-\frac{La}{\lambda}\right) + 1. \qquad (1)$$

Here, a is the lattice constant, L is the number of the $SrTiO_3$ barrier layers, and λ is the mean free path of photoelectrons. The excellent agreement between the experimental and simulation results indicates the successful digital control of the $SrTiO_3$ barrier layer in the present double QW structures.

The excellent agreement also demonstrates the formation of a chemically abrupt interface within the experimental margins. The results of these core-level analyses are consistent with the invariance of the QW states between the surface (vacuum/$SrVO_3$ interface) and the interface ($SrTiO_3$/$SrVO_3$), as shown in Supplementary Fig. 25. From these spectroscopic results, it is naturally concluded that the prepared double QW structures feature atomically and chemically abrupt $SrVO_3$/$SrTiO_3$ interfaces with interdiffusion length of less than 0.2 nm, and the possible intermixing has a negligible influence on our ARPES observations.

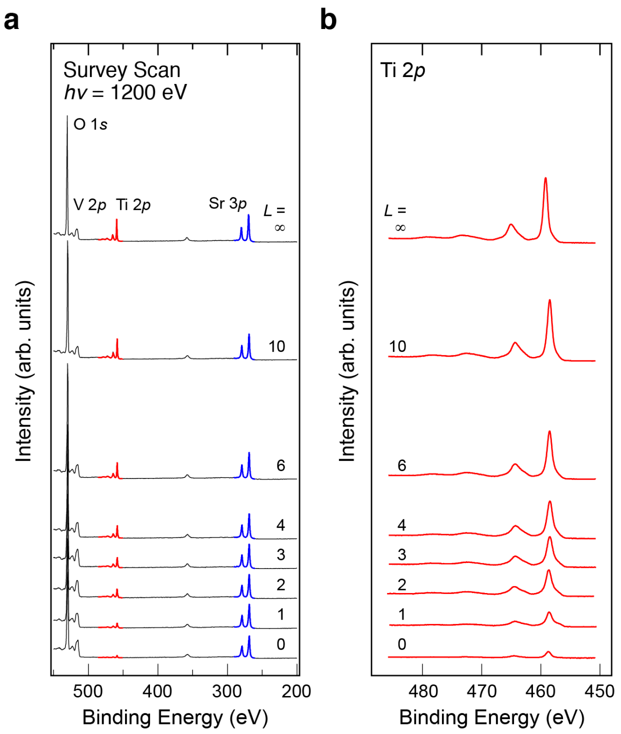

Supplementary Figure 5: Core-level analysis for $V_2T_LV_6$ double QW structures. **a**, Core-level spectra of $SrVO_3$ (2 ML)/$SrTiO_3$ (L ML)/$SrVO_3$ (6 ML)/ $SrTiO_3$ substrate structures with varying $SrTiO_3$ barrier layer thickness L. **b**, Ti 2p core level in an expanded energy scale. Note that all spectra are normalized with the intensity of the Sr 3p core level.

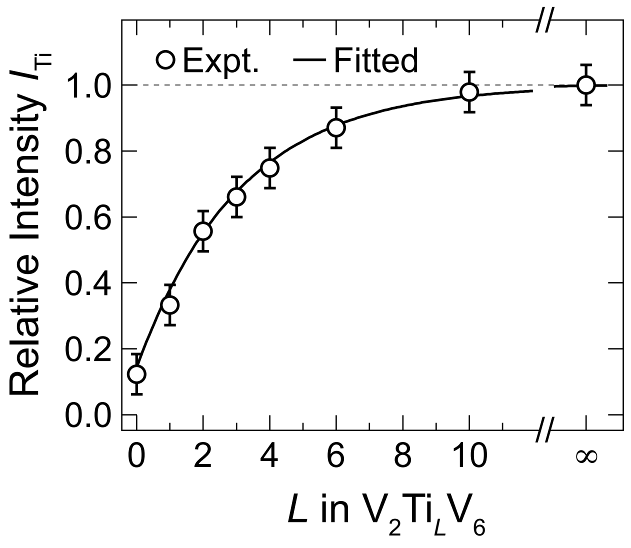

Supplementary Figure 6: Digital control of $SrTiO_3$ layers in $V_2T_LV_6$ double QW structures.

Plots of the intensity of Ti 2*p* core levels as a function of *L* (open circles with error bars), together with the simulated curve derived from Supplementary Eq. (1) (a black line). Error bars reflect the uncertainties originating from the energy resolution and statistics of data.

5.3 Chemical analysis for core-level spectra

The almost constant chemical states in the $SrVO_3$ (2 ML)/$SrTiO_3$ (*L* ML)/$SrVO_3$ (6 ML)/ $SrTiO_3$ substrate structures irrespective of varying *L* are confirmed by the chemical analysis of respective core levels as shown in Supplementary Fig. 7. The shape of the core levels remains almost identical, although some broadenings due to the metallization of the top 2-ML $SrVO_3$ layer are observed with reducing *L*. More detailed information on these spectra can be obtained by curve fitting, as shown in Supplementary Fig. 8.

For Ti 2*p* core levels, the shape reflects the chemical states in the $SrTiO_3$ barrier layer for $L = 2$, 4, 6, and 10 or those in $SrTiO_3$ single-crystal substrates for $L = \infty$ [$SrVO_3$ (2 ML)/ $SrTiO_3$ substrate structures] owing to the elemental selectivity of the x-ray photoemission (XPS) measurements. The line shape of the Ti 2*p* core level maintains its original Ti^{4+} feature, indicative of the invariance of the chemical environments even at the interface. Although the core levels remain almost identical, the width of the main peak at 458.5 eV is slightly broadened with reducing *L*. The slight broadening of the main peak may be due to the metallization of the top $SrVO_3$ layer through the resonant tunneling (RT) effects. In addition, an almost exact match between $L = 10$ and ∞ indicates that there is no fundamental difference between the $SrTiO_3$ barrier layers and $SrTiO_3$ substrates.

In contrast to the Ti 2*p* core levels, the other ones mainly reflect the chemical states of the top 2-ML $SrVO_3$ layer owing to the probing depth of the XPS measurements. For V 2*p* core levels, the complicated final-state effects[11] and some contributions from the bottom $SrVO_3$ layers make any quantitative analysis difficult, although the existence of V^{5+} states at 517.5–518 eV due to the surface oxidation[12–14] is hardly seen in the spectra. For this reason, the V 2*p* core levels were excluded from the curve fitting analysis. For Sr 3*d* core level, a close inspection reveals that slight changes occur in line shapes of $L = 2$ and 4, reflecting the metallization of the top $SrVO_3$ layer: with reducing *L*, an asymmetric parameter (α) of Doniach-Sunjic line shape increases. These behaviors are consistent with the ARPES results shown in Fig. 2 in the main text, providing

further support for metallization of the top $SrVO_3$ layer due to RT effects.

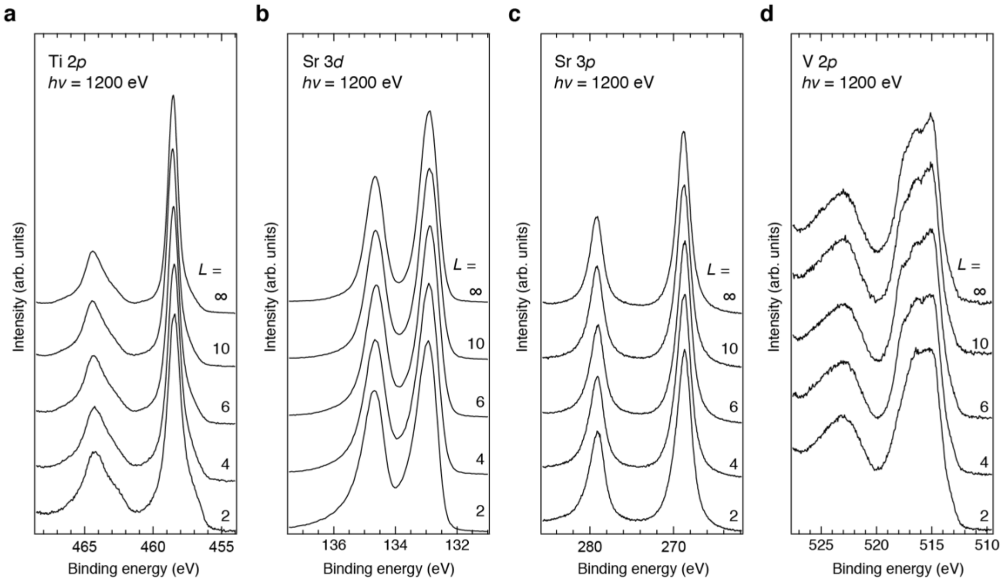

Supplementary Figure 7: Chemical analysis for core-level spectra of $V_2T_LV_6$ double QW structures. (**a**) Ti 2*p*, (**b**) Sr 3*d*, (**c**) Sr 3*p*, and (**d**) V 2*p* core levels for $SrVO_3$ (2 ML)/$SrTiO_3$ (*L* ML)/$SrVO_3$ (6 ML)/ $SrTiO_3$ substrate structures with varying $SrTiO_3$ barrier layer thickness *L*. Note that $L = \infty$ corresponds to $SrVO_3$ (2 ML)/ $SrTiO_3$ substrates. All spectra are normalized with the intensity of the prominent peaks. Note that the Ti 2*p*, Sr 3*p*, and V 2*p* core levels are the same as shown in Supplementary Fig. 5.

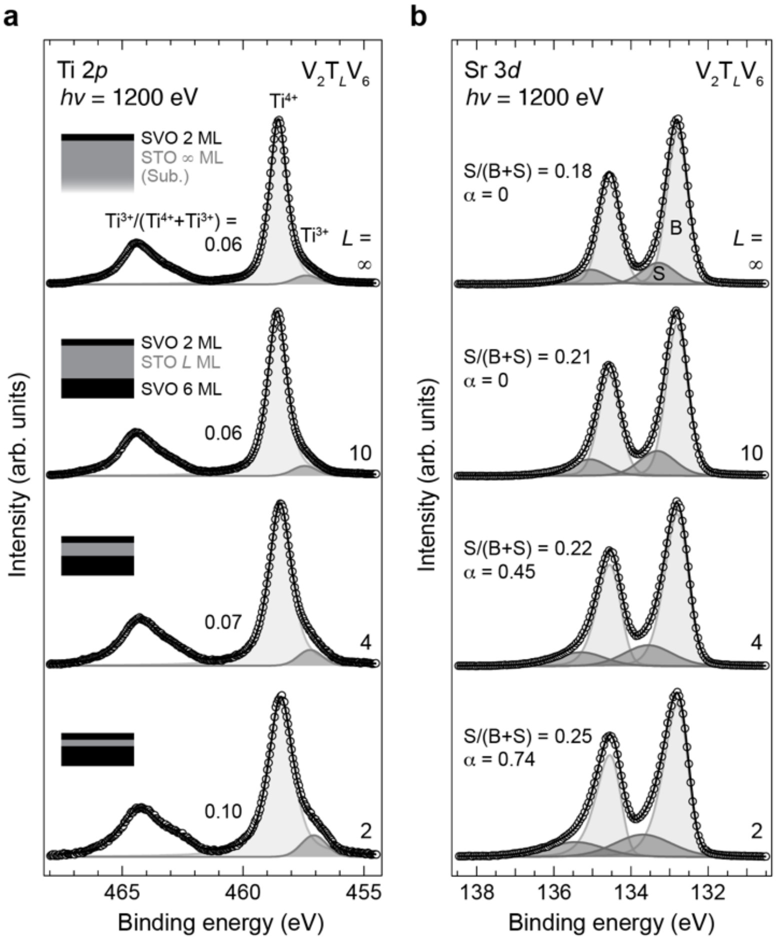

Supplementary Figure 8: Curve fitting analysis for core-level spectra of $V_2T_LV_6$ double QW structures. (**a**) Ti 2*p* core-level spectra (open circles) and their decompositions (solid lines) for $SrVO_3$ (2 ML)/$SrTiO_3$ (*L* ML)/$SrVO_3$ (6 ML)/ $SrTiO_3$ substrate structures. The curve fitting results for the Ti-2$p_{3/2}$ states are presented, where the components of the Ti^{4+} (458.5 eV) and Ti^{3+} (457–457.5 eV) states[14,15] are indicated by different hatching. The fractions of Ti^{3+} states [Ti^{3+}/(Ti^{4+}+Ti^{3+})] are denoted for each core level. (**b**) Sr 3*d* core-level spectra (open circles) and their decompositions (solid lines). The bulk (B) and surface (S) components[12,16,17] are indicated by different hatching. The fractions of surface components [S/(S+B)] and the asymmetric parameters (α) of the Doniach-Sunjic line shape for the bulk component are denoted for each core level.

5.4 Characterization of double QW structures by HAADF-STEM measurements

Supplementary Figure 9 shows a cross-sectional high-angle annular dark-field scanning transmission electron microscope (HAADF-STEM) image of the amorphous $SrTiO_3$/ $SrVO_3$ (20 ML)/ $SrTiO_3$ (2 ML)/ $SrVO_3$ (6 ML)/ Nb:$SrTiO_3$ substrate structure. The HAADF-STEM measurement confirms the coherent growth of the $SrVO_3$ and $SrTiO_3$ layers on the $SrTiO_3$ substrate without the formation of any dislocations.

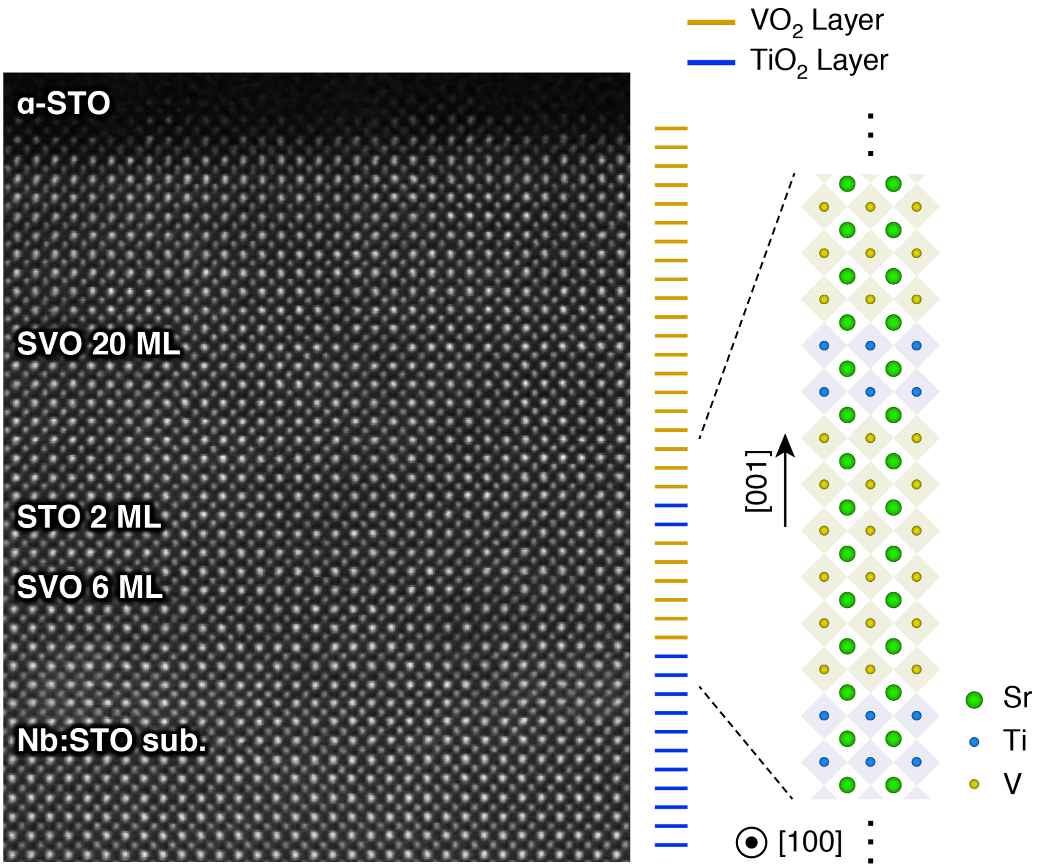

Supplementary Figure 9: HAADF-STEM characterization for double QW structures. A cross-sectional HAADF-STEM image along the [100] direction of the amorphous $SrTiO_3$/ $SrVO_3$ (20 ML)/ $SrTiO_3$ (2 ML)/ $SrVO_3$ (6 ML)/ Nb:$SrTiO_3$ (001) substrate structure. The right-hand panel depicts the schematic for the STEM image. Note that it is hard to distinguish between the $SrVO_3$ and $SrTiO_3$ layers, because there is no detectable difference in atomic weight Z between Ti (Z = 22) and V (Z = 23) ions, and both oxides share a common A-site composition (SrO). Thus, the atomic layer arrangement shown in the right-hand panel is deduced by counting the atomic layers from the top.

Supplementary Note 6. "*In situ* photoelectron spectrometer – laser molecular beam epitaxy" system

Supplementary Figure 10 shows the photograph of our "*in situ* photoelectron spectrometer – laser molecular beam epitaxy (MBE)" system, in which a high-resolution angle-resolved photoemission (ARPES) apparatus (VG-Scienta SES2002 hemispherical electron analyzer) is connected to laser-MBE equipment in an ultrahigh vacuum (UHV). This system consists of four interconnected chambers: sample entry, sample preparation, laser MBE, and photoemission (PES). The PES chamber is connected to the beamline. The four chambers are connected to each other under UHV conditions, and each chamber can be isolated using gate valves. A typical sequence of sample growth and measurements is as follows. First, a substrate mounted on a sample holder is loaded into the sample entry chamber and transferred to the laser MBE chamber via the sample preparation chamber. Oxide QW structures are grown on the substrate by pulsed laser deposition while monitoring the intensity oscillation of reflection high-energy electron diffraction (RHEED). The fabricated QW structures are then transferred back to the sample preparation chamber, where their surface structure and surface cleanness can be characterized by low-energy electron diffraction (LEED) and Auger electron spectroscopy (AES). After surface characterization, the sample is moved with a transfer rod into the photoemission measurement stage. The sample transfer is carefully operated under a UHV of 10^{-10} Torr to avoid contamination of the sample surface during transfer. Such *in situ* photoemission analysis is crucial for investigating the electronic structure of QW structures, as well as oxide thin films and heterostructures.

We demonstrate the importance of in-vacuum sample transfer and the cleanliness of our *in situ* transfer of samples. Supplementary Figure 11 shows a survey scan of the x-ray photoemission (XPS) spectra of the QW structures just before ARPES measurements. No detectable C 1*s* signal is observed on the surface of the in-vacuum transferred film, indicating the absence of contaminations such as carbon oxides at the measured surface.

This system has been installed at the new undulator beamline 2A MUSASHI (Multiple Undulator beamline for Spectroscopic Analysis on Surface and HeteroInterface) as an endstation. At the BL-2A MUSASHI, we performed both vacuum ultraviolet (VUV: 30–300 eV for ARPES) and soft x-ray (SX: 250–2000 eV for XPS) spectroscopic measurements at the same time, for the same

grown sample, with the same experimental setup. This approach ensures the quality of the experimental data and offers numerous benefits to the field of surface science, enabling us to obtain comprehensive and highly reproducible information regarding the electronic and chemical structures of oxide nanostructures fabricated by laser MBE. For example, the chemical states and thicknesses of each layer of QW structures are characterized by XPS (see Supplementary Note 5), confirming the quality of the heterostructures. Subsequently, their band structures are investigated by ARPES using VUV light. Note that all ARPES measurements were conducted after confirming the high surface cleanliness required for ARPES measurements.

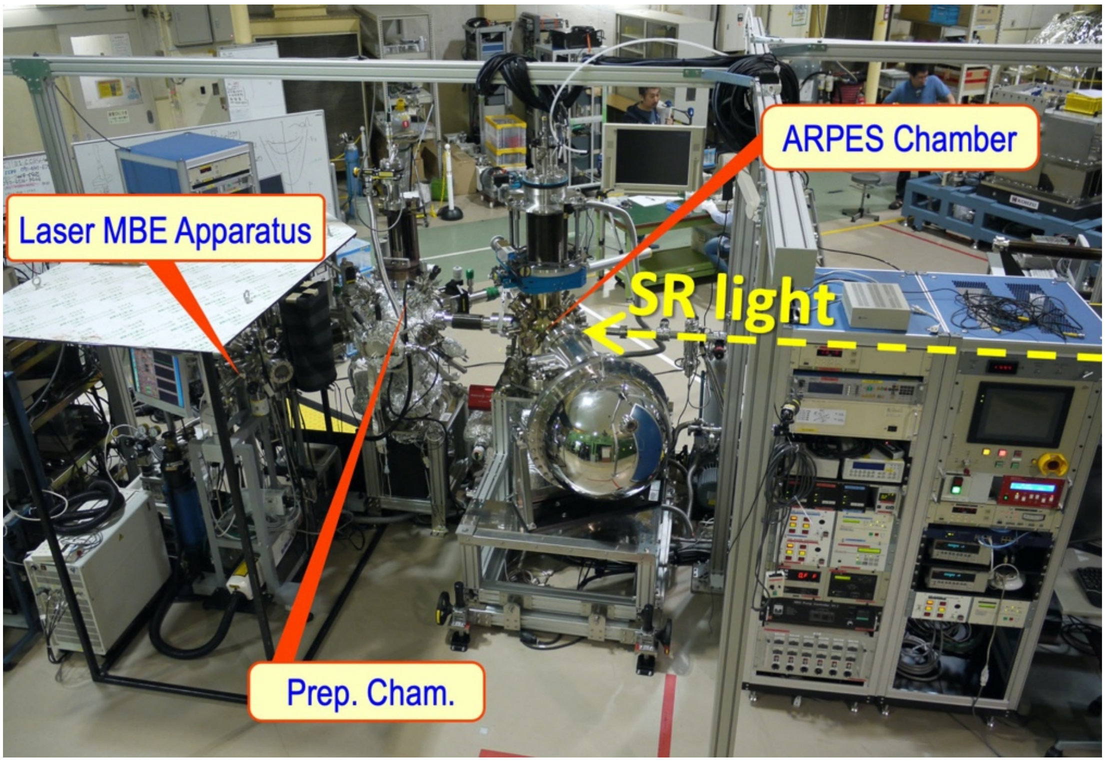

Supplementary Figure 10: Photograph of "*in-situ* photoemission – Laser MBE system". This system is installed as an end station of BL-2A MUSASHI.

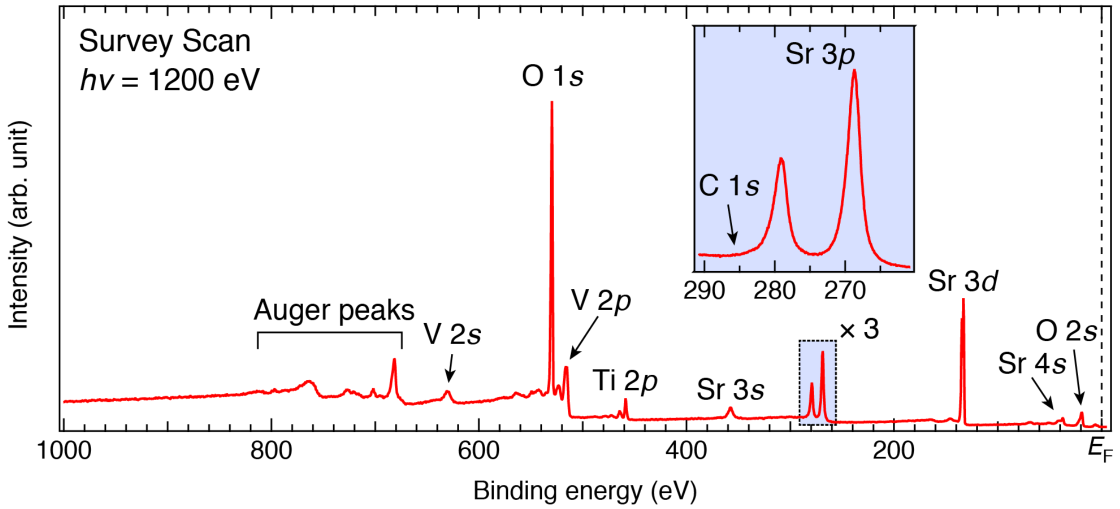

Supplementary Figure 11: Survey scan of the *in-situ* transferring $V_2T_2V_6$ double QW structures. No detectable C 1*s* signal is observed, indicating that contaminations such as carbon oxides are absent at the measured surface.

Supplementary Note 7. Experimental geometry in the present ARPES measurement

Supplementary Figure 12a shows the sketch of our experimental geometry for *in situ* ARPES measurements. The incident synchrotron radiation beam and the outgoing photoelectrons entering the analyzer slit define the emission plane, which is horizontal in this case. The light polarization (horizontal or vertical) is referred to this emission plane. The reciprocal space along the X- and Y-axis directions is explored by varying the angles of θ and φ, respectively.

Supplementary Figures 12b and 12d show the ARPES intensity maps along the Γ-X direction for a $SrVO_3$ film of 8-ML thickness taken with linear horizontal (LH) and linear vertical (LV) polarizations, respectively, via comparison with the corresponding results of the tight-binding calculation in the mass renormalization scheme for each $3d$ t_{2g} orbital[3] (Supplementary Fig. 12c). A significant polarization dependence is clearly observed. Owing to the dipole selection rules for the present experimental setup, the ARPES intensities of the d_{zx}-derived subbands are dominant in the LH mode (Supplementary Fig. 12b), whereas the d_{xy}- and d_{yz}-derived subbands in the LV mode (Supplementary Fig. 12d). In other words, the polarization-dependent ARPES enables the determination of the subbands of d_{zx} states and d_{xy}/d_{yz}-states separately.

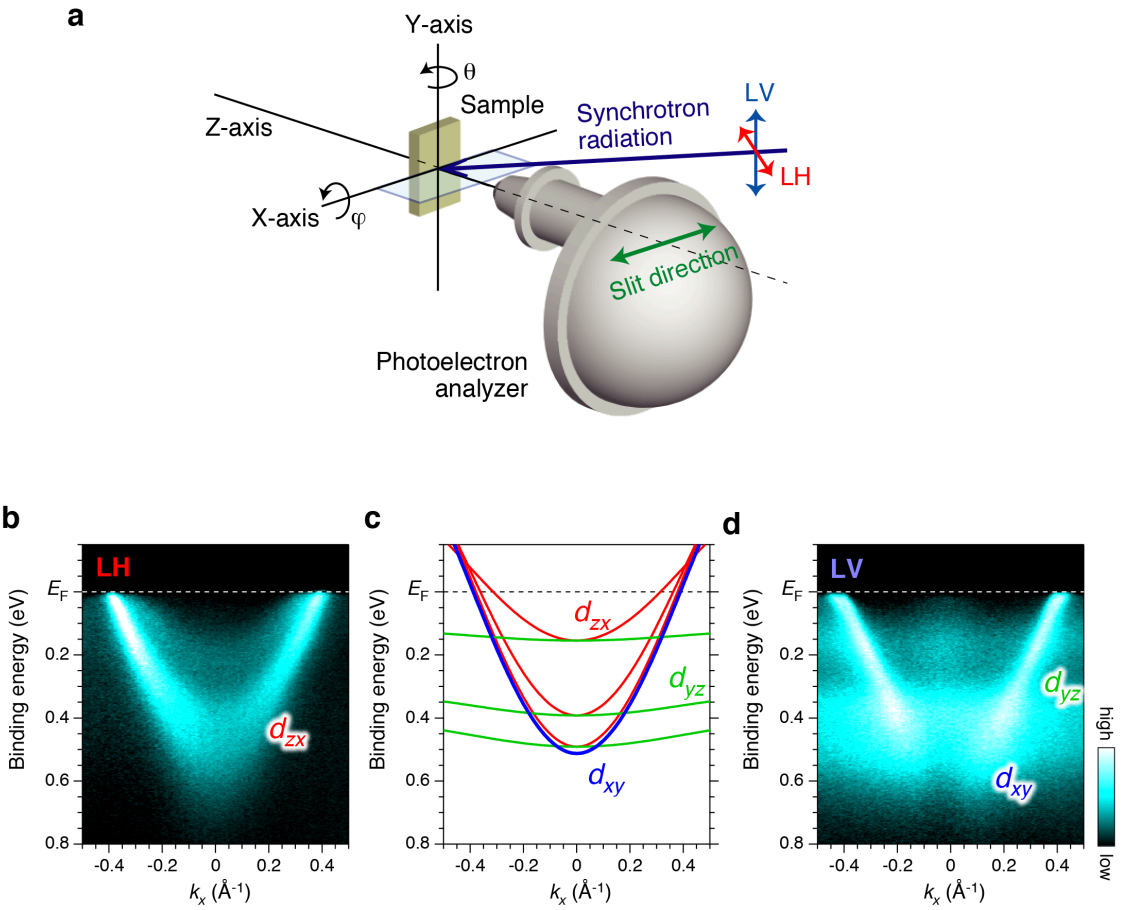

Supplementary Figure 12: Experimental geometry for *in situ* ARPES measurements and polarization-dependent ARPES results. **a**, Sketch of the experimental geometry at the BL-2A MUSASHI of the Photon Factory, KEK. LH and LV denote linear-horizontal and linear-vertical polarizations, respectively. In this setup, the incoming photon momentum is coplanar to the entrance slit (photoelectron detection) direction of the photoelectron analyzer. **b**,**d**, ARPES intensity maps taken at a photon energy of 88 eV along the Γ-X direction for an 8-ML $SrVO_3$ film with LH (**b**) and LV (**d**) polarized lights. **c**, Tight-binding calculation result for the 8-ML $SrVO_3$ (Supplementary Ref. 3). The blue, green, and red curves represent the d_{xy}, d_{yz}, and d_{zx} subbands, respectively. It is evident that the ARPES intensity of the d_{zx}- (d_{yz}/ d_{xy}-) derived subbands becomes dominant in the LH (LV) mode.

Supplementary Note 8. Analysis of ARPES spectra

8.1 ARPES images

Supplementary Figure 13 shows the ARPES intensity plots in the energy–momentum (*E*–*k*) space of $V_6T_2V_2$ and $V_2T_2V_6$ double QW structures, together with their curvature intensity plots[18]. The intensity modulation of the ARPES images between the left and right sides with respect to the centerline (k_x = 0 $Å^{-1}$) is due to pronounced matrix-element effects. In order to show the band structure more clearly, we display the images enclosed by the dotted squares in the main text (Fig. 3). Note that we carefully checked that the band dispersion itself is almost the same between the ARPES intensity plots and the curvature plots by picking up the peak position from the raw data (Supplementary Fig. 14).

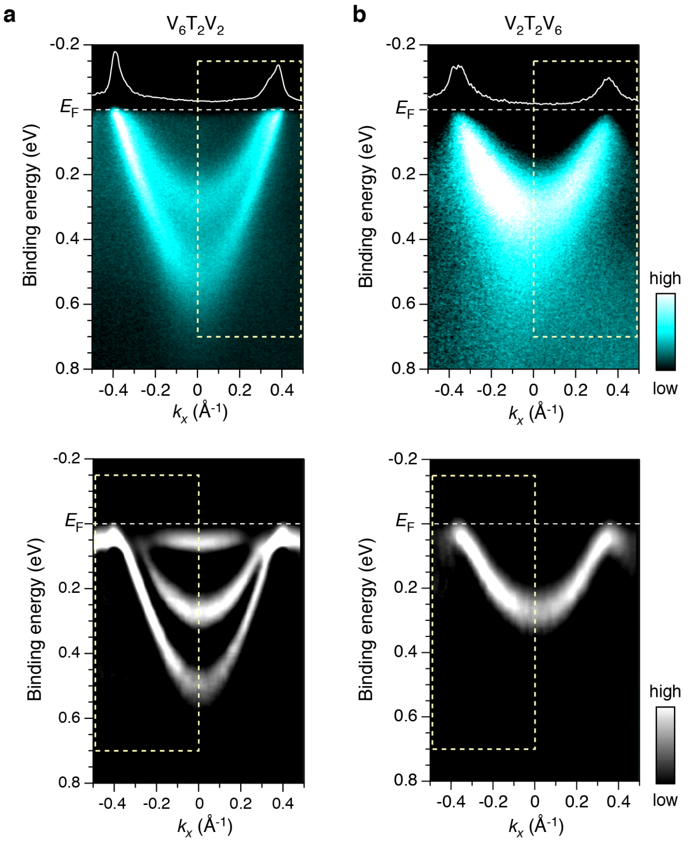

Supplementary Figure 13: ARPES-image analysis for $V_6T_2V_2$ and $V_2T_2V_6$ double QW structures. Intensity plots of the ARPES spectra taken along the Γ-X direction for $V_6T_2V_2$ (**a**) and $V_2T_2V_6$ (**b**) double QW structures (upper panels) and their curvature intensity plots (lower

panels). The intensity modulation is due to pronounced matrix-element effects in the ARPES measurements. The images enclosed by the dotted squares are displayed in the main text (Fig. 3). The momentum distribution curves (MDCs) at the Fermi level (E_F), integrated over an energy window of 20 meV, are also shown in the respective ARPES images.

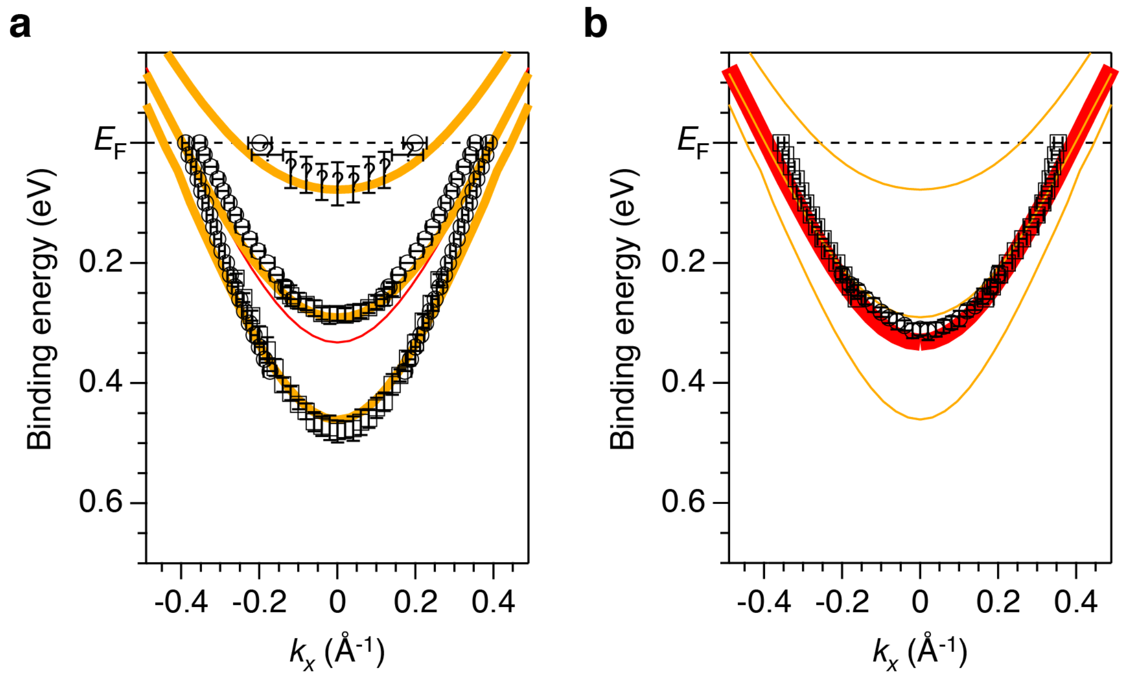

Supplementary Figure 14: ARPES-peak plots for $V_6T_2V_2$ and $V_2T_2V_6$ double QW structures. Plots of peak positions (data markers) determined from ARPES data along the Γ-X direction (the upper panels of Supplementary Fig. 13) for $V_6T_2V_2$ (**a**) and $V_2T_2V_6$ (**b**) double QW structures in comparison with the corresponding DFT results (solid lines). The peak positions solidly determined from the MDCs and energy distribution curves (EDCs) are denoted by the open circles and open squares, respectively. Question marks denote the energy positions of weak structures. The solid lines show the DFT results for the d_{zx} quantization states (Fig. 3 in the main text and Supplementary Fig. 26). Error bars reflect the uncertainties originating from the energy resolution and the standard deviation in the peak positions of MDCs and EDCs.

8.2 Momentum distribution curves for a series of $V_2T_LV_6$ double QW structures

Supplementary Figure 15 shows the ARPES images of a series of $V_2T_LV_6$ double QW structures. The data are the same as those in Fig. 2 in the main text but are displayed in the expanded energy scale up to 0.8 eV. The momentum distribution curves (MDCs) at the Fermi level (E_F), integrated over an energy window of 20 meV, are shown in the respective ARPES images. It is evident that the Fermi surface emerges for $V_2T_2V_6$ double QW structures. The emergence of the Fermi surface is further confirmed by the fact that the MDC is well fitted by two Lorentzian functions, as shown in Supplementary Fig. 16.

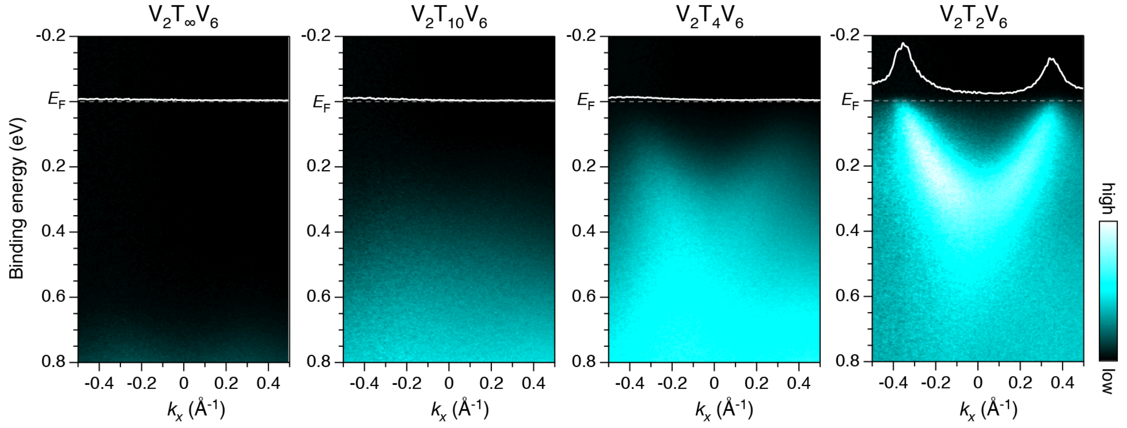

Supplementary Figure 15: ARPES intensity plots for a series of $V_2T_LV_6$ double QW structures. Note that the data are the same as those in Fig. 2 in the main text. The MDCs at E_F integrated over an energy window of 20 meV are overlaid in the respective images.

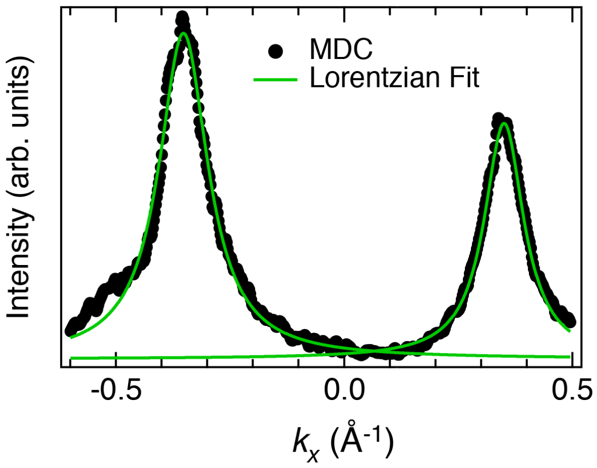

Supplementary Figure 16: MDC at E_F for the $V_2T_2V_6$ double QW structure. The MDC is well fitted to two Lorentzian functions (green curves).

8.3 Momentum distribution curves for band crossing E_F

Supplementary Figure 17 shows the MDCs in the energy range from 0.1 eV to -0.04 eV for a series of $V_2T_LV_6$ double QW structures to confirm the emergence of the subband crossing over E_F. For $V_2T_2V_6$, it is clearly observed that the MDC peaks, which correspond to the band dispersion in the rightmost panel of Fig. 2 in the main text (Supplementary Figs. 13b, 14b, and the rightmost panel of Supplementary Fig. 15), evidently cross E_F, indicating the metallization of the $SrVO_3$ top-QW layer. Since MDCs and EDCs present essentially identical band dispersions, the MDC analysis provides further support for the emergence of the metallic band that crosses E_F. Note that we separately determined the band dispersion by picking up the peak positions of the MDCs and EDCs (see Fig. 2b in the main text), and confirmed that the band dispersions obtained from the two different types of curves were identical.

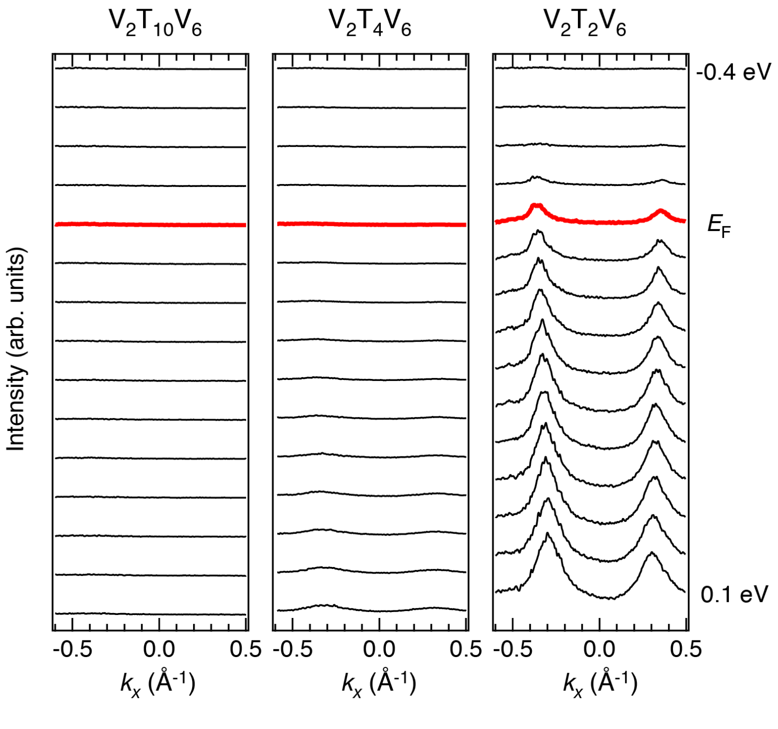

Supplementary Figure 17: MDCs for band crossing E_F. MDCs for the $V_2T_LV_6$ double QW structures with L = 10, 4, and 2 (from left). The intensities of the MDCs are normalized to the incident photon flux. Thick red lines indicate the MDCs at E_F.

Supplementary Note 9. Spectral weight transfer from the lower Hubbard band to the coherent band near E_F

Supplementary Figure 18 shows the valence-band spectra for the $V_2T_LV_6$ double QW structures with varying L from ∞ to 2. These spectra exhibit remarkable and systematic changes. The valence band mainly consists of three structures[8]: two prominent O 2p derived structures exist at binding energies of 3.0–9.0 eV, whereas a characteristic structure emerges near E_F. The structure near E_F was assigned to the V 3d states on the basis of V 2p-3d resonant photoemission spectra. According to the previous results on $SrVO_3$ QW structures[8], a sharp peak located precisely at E_F corresponds to the coherent part (quasiparticle peak), while a relatively broad peak centered at 1.0–1.5 eV the incoherent part (the remnant of the lower Hubbard band). With decreasing the barrier layer thickness, the leading edge of the V 3d states clearly shifts to E_F, and the spectral weight at E_F seems to increase from $V_2T_4V_6$. Eventually, the sharp peak evolves at E_F for the $V_2T_2V_6$ double QW structure, indicating the metallization of the $SrVO_3$ top-QW layer.

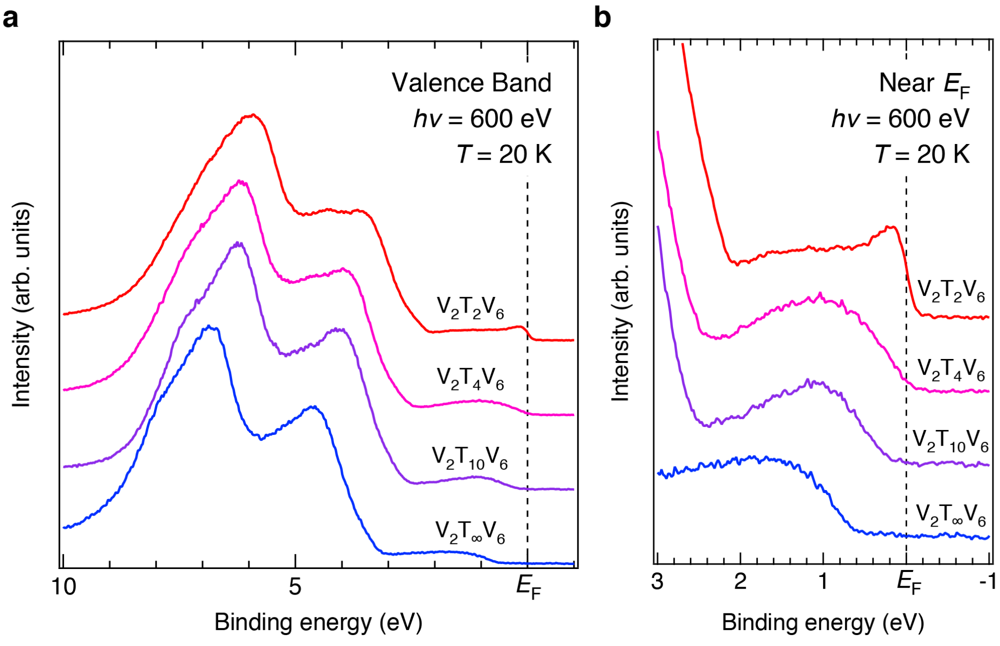

Supplementary Figure 18: Valence-band spectra for $V_2T_LV_6$ double QW structures. Angle-integrated PES spectra for valence-band (**a**) and near-E_F (**b**) region for $V_2T_LV_6$ double QW structures with varying L from ∞ to 2. These spectra were taken at a photon energy $h\nu = 600$ eV and a temperature of 20 K.

Supplementary Note 10. Series of ARPES images for $V_2T_LV_6$ double QW structures with reducing L from ∞ to 1

Supplementary Figure 19 presents a series of ARPES results for the $V_2T_LV_6$ double QW structures with varying the $SrTiO_3$ barrier layer thicknesses (L = 1, 2, 3, 4, 10, and ∞ ML). Here, the series of ARPES images are normalized to the incident photon flux; hence, the color scale reflects the change in spectral weight as a function of L. The metallization of the top QW with reducing L is evidenced by the appearance of a parabolic band at the Γ point near the Fermi level (E_F). As predicted by the DFT calculations (Supplementary Fig. 27), the metallic band near E_F emerges gradually. Furthermore, the band dispersion itself remains unchanged, whereas its intensity is enhanced with reducing L. The spectral behavior strongly suggests the occurrence of the resonant-tunneling (RT) driven MIT.

Supplementary Figure 20 shows the plot of the quasiparticle peak (MDC peak) intensity at E_F as a function of L. A steep attenuation of the intensity with increasing L is observed, reflecting the nature of the tunneling effect. By fitting the data using an exponential decay function of $\exp(-La/\lambda)$ where a is the lattice constant of $SrTiO_3$, we determined the characteristic length scale of the barrier layer required for the RT-induced MIT: λ = 0.59 nm (~1.5 ML).

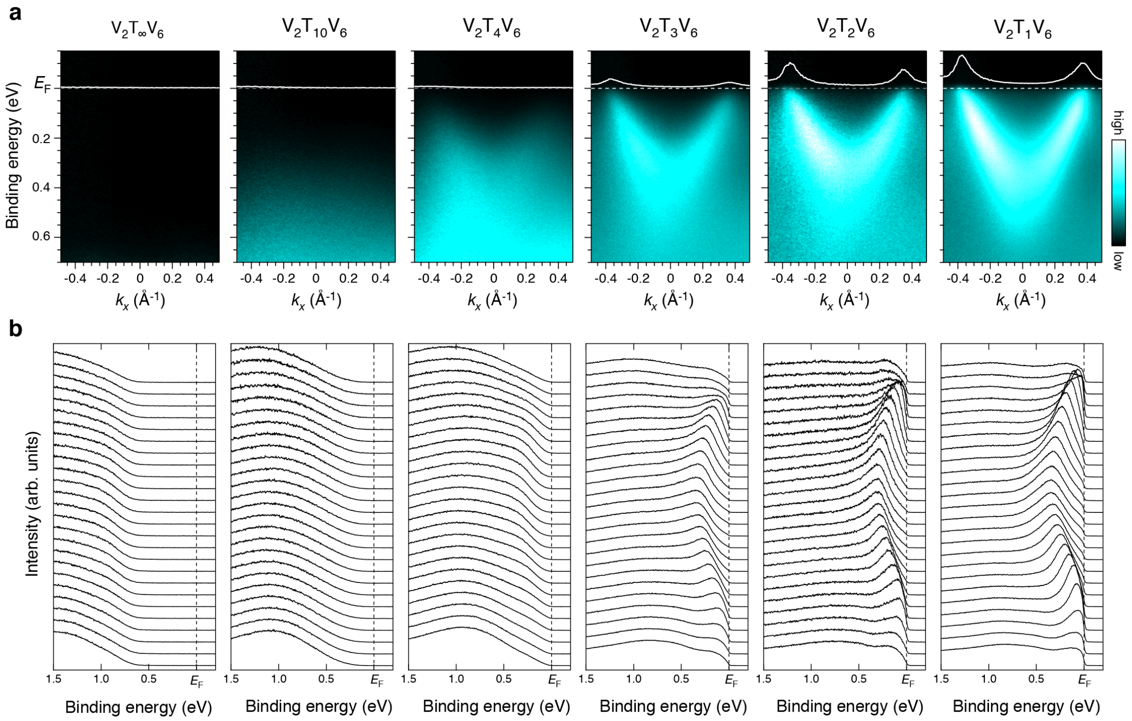

Supplementary Figure 19: Series of ARPES images for $V_2T_LV_6$ double QW structures. **a**, Respective ARPES images for $V_2T_LV_6$ double QW structures as L decreases from ∞ to 1, where the ARPES images for L = 2, 4, 10, and ∞ are the same as those in Fig. 2 in the main text. The MDCs at E_F, integrated over an energy window of 20 meV, are overlaid in the respective images. Note that the series of ARPES spectra are normalized to the incident photon flux, and the normalized intensity is given by a color scale shown on the right-hand side. **b**, EDCs corresponding to the respective ARPES images. The broad non-dispersive feature around 1.0 eV is the lower Hubbard band.

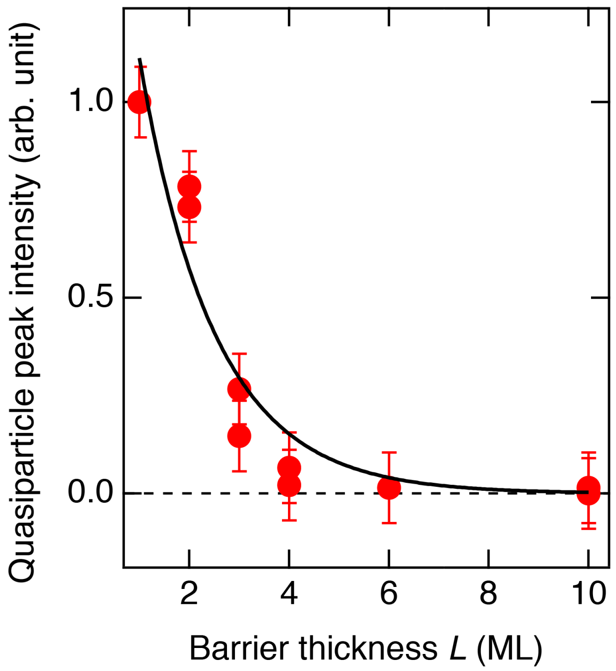

Supplementary Figure 20: Attenuation of the quasiparticle intensity in $V_2T_LV_6$ double QW structures. The plot of quasiparticle-peak intensity at E_F as a function of L. The experimental points are fitted to an exponential decay function (a solid line). Error bars reflect the uncertainties originating from the energy resolution and statistics of data.

Supplementary Note 11. RT-driven MIT in $V_2T_LV_5$ double QW structures

11.1 ARPES images for a series of $V_2T_LV_5$ double QW structures

Supplementary Figure 21 shows the ARPES images for a series of $V_2T_LV_5$ double QW structures, together with an ARPES image of $V_2T_2V_6$ (the same as that in Fig. 2 in the main text) for comparison. The momentum distribution curves (MDCs) at the Fermi level (E_F), integrated over an energy window of 20 meV, are shown in the respective ARPES images. All ARPES images are normalized to the incident photon flux, and the normalized intensity is given by a color scale shown on the right-hand side. For a series of $V_2T_LV_5$ double QW structures, the barrier-thickness dependence resembles to that of $V_2T_LV_6$ (Fig. 2 in the main text): as the $SrTiO_3$ barrier layer becomes thinner, a faint dispersive feature emerges near E_F. Eventually, a metallic band whose dispersion crosses E_F is clearly visible in the $V_2T_2V_5$ double QW structures. Furthermore, the metallic band dispersion of $V_2T_2V_5$ is almost identical to that of $V_2T_2V_6$. These results indicate the metallization of the top 2-ML $SrVO_3$ layer of $V_2T_2V_5$, as in the case of $V_2T_2V_6$.

Interestingly, a closer look reveals that the intensity of the coherent band in $V_2T_2V_5$ is much weaker than that in $V_2T_2V_6$. This intensity reduction in the metallic band may be attributable to the energy offset of the corresponding QW states (between $n = 2$ in the bottom QW structure and $n = 1$ in the top one, as shown in the structure plot in Supplementary Fig. 1), suggesting that the MIT is derived from the resonant tunneling in the double QW structures.

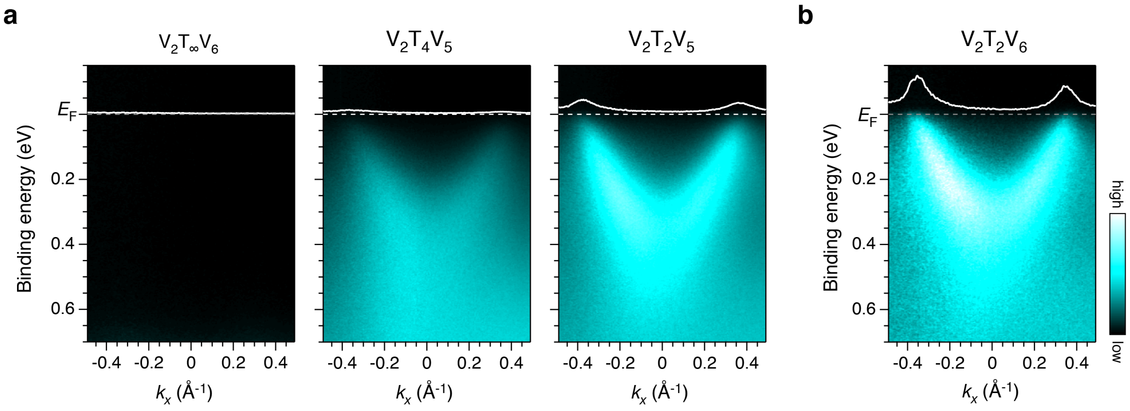

Supplementary Figure 21: ARPES images for $V_2T_LV_5$ double QW structures. **a**, Series of ARPES images for $V_2T_LV_5$ double QW structures with reducing L from ∞ to 2, together with an ARPES image of $V_2T_2V_6$ (**b**) for comparison. The MDCs at E_F are overlaid in the respective

images. Note that the series of ARPES spectra are normalized to the incident photon flux, and the normalized intensity is given by a color scale shown on the right-hand side.

11.2 ARPES images for $V_2T_2V_5$ and $V_5T_2V_2$

Supplementary Figure 22 shows the ARPES intensity plots and their corresponding curvature intensity plots in the energy–momentum (E–k) space of $V_5T_2V_2$ and $V_2T_2V_5$ double QW structures in the same manner as in Supplementary Fig. 13. It should be noted that there is no detectable difference in the dispersion of the metallic band appearing at the top 2-ML QW between $V_2T_2V_5$ and $V_2T_2V_6$ (Supplementary Fig. 13), reflecting the resonant tunneling nature of the metallization.

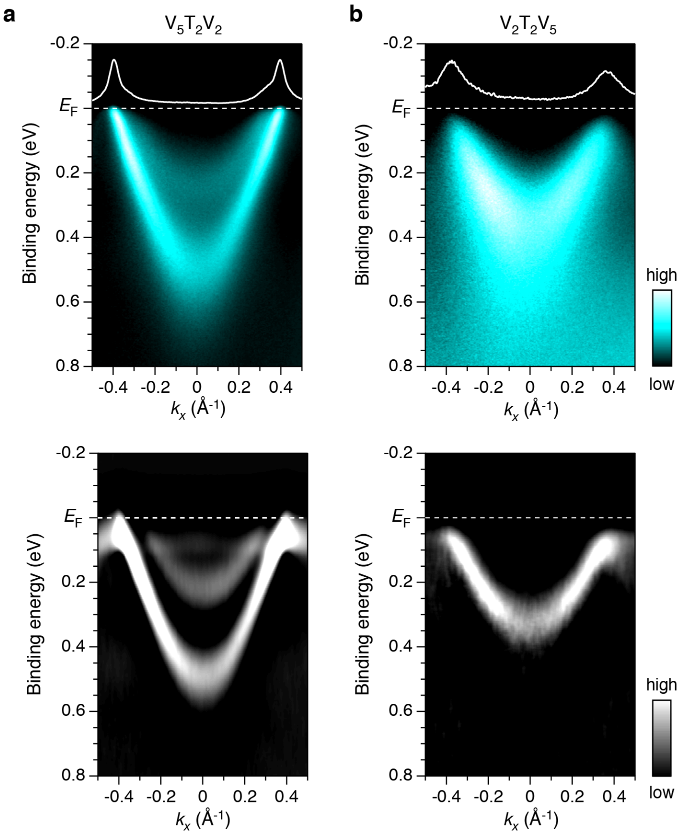

Supplementary Figure 22: ARPES-image analysis for $V_5T_2V_2$ and $V_2T_2V_5$ double QW structures. Intensity plots of the ARPES spectra taken along the Γ-X direction for $V_5T_2V_2$ (**a**) and $V_2T_2V_5$ (**b**) double QW structures (upper panels) and their curvature intensity plots (lower panels). The intensity modulation is due to pronounced matrix-element effects in the ARPES measurements. The MDCs at E_F are also shown in the respective ARPES images.

Supplementary Note 12. Contributions from bottom QW states in ARPES images

Supplementary Figure 23 shows a series of ARPES images for the $V_2T_2V_6$ double QW structures taken at different photon energies in the range of 88–1013 eV. As can be seen in Supplementary Fig. 23, there are not any fundamental differences in the observed band structure due to the change in photon energies, except for the peak broadening caused by the poor energy resolution at higher photon energies. These results indicate that the contribution from the bottom QW states is negligible and the observed metallic states are at the top 2-ML $SrVO_3$ layer.

The negligible contribution from the bottom QW states is further confirmed by ARPES images measured for 4-ML $SrTiO_3$/ 6-ML $SrVO_3$ bilayer structures, as shown in Supplementary Fig. 24. Using the bilayer, we can estimate the contribution from the bottom metallic QW states to the ARPES results on the 2-ML $SrVO_3$/ 2-ML $SrTiO_3$/ 6-ML $SrVO_3$ double QW structures. Since these ARPES images shown in Supplementary Fig. 24 are normalized to the incident photon flux, it is evident that the contribution from the buried metallic QW states is negligible in the present ARPES data taken at $h\nu = 88$ eV.

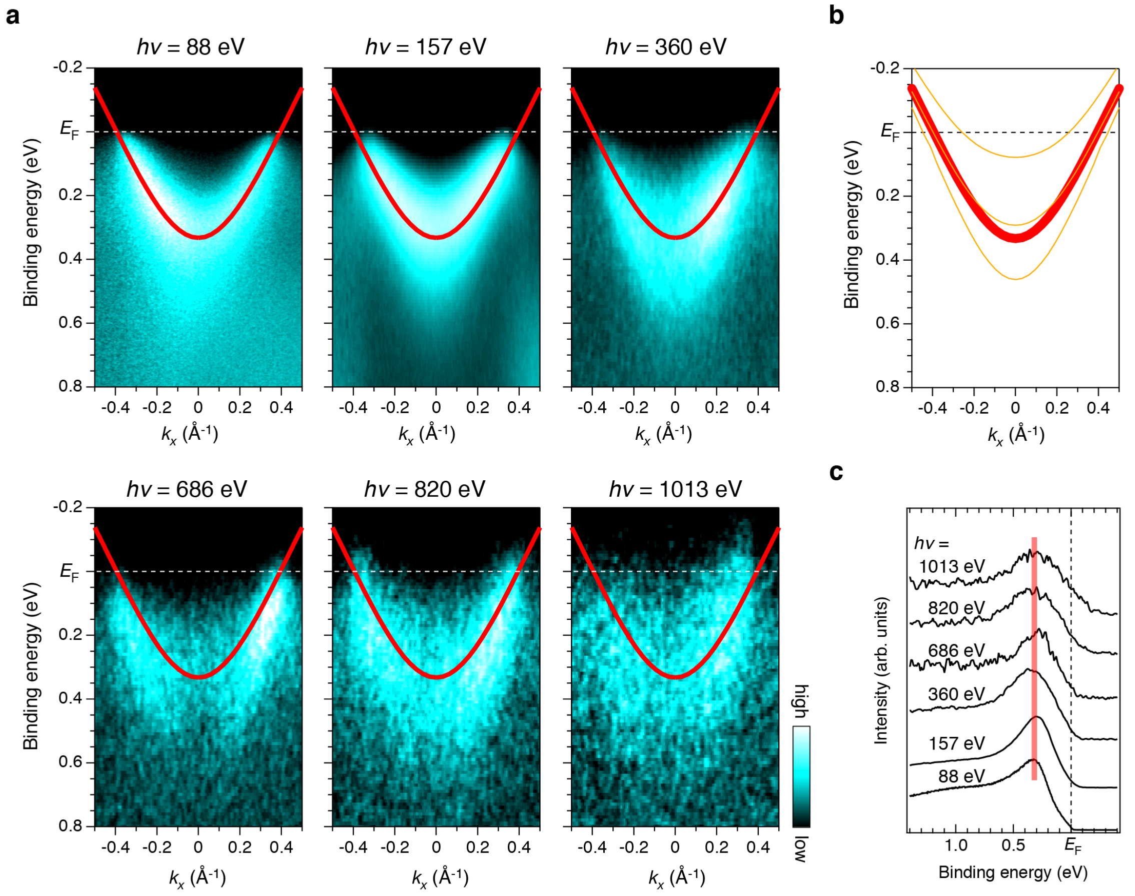

Supplementary Figure 23: Photon-energy dependence of ARPES images of $V_2T_2V_6$ double QW structures. **a**, ARPES images of $V_2T_2V_6$ double QW structures taken at different photon energies in the range of 88–1013 eV, where the result of the DFT calculation for the top QW states (n = 1 in 2-ML $SrVO_3$ QW states) is overlaid as a solid red line. **b**, The DFT calculation results for the top (a solid red line) and bottom (solid orange lines) QW structures that are shown in Supplementary Fig. 14b. **c**, Comparison of ARPES spectra taken at the Γ point under the different photon energies.

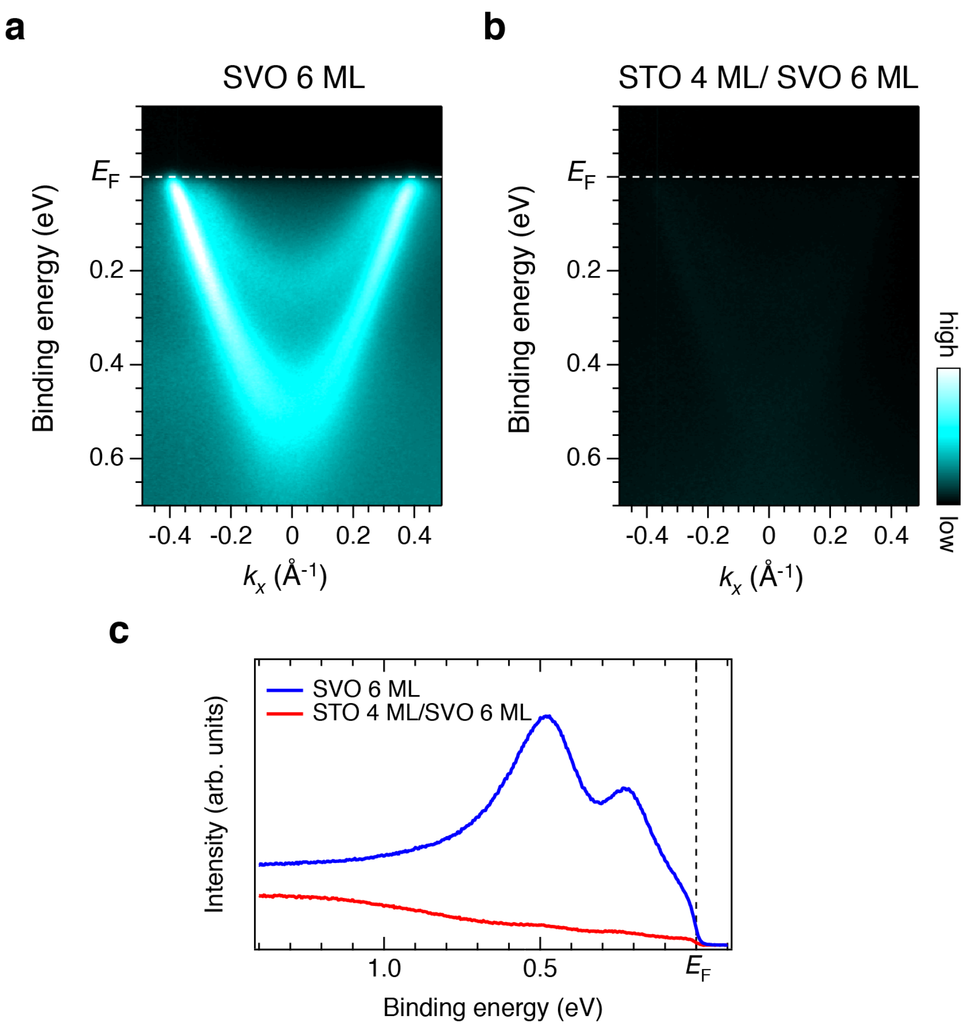

Supplementary Figure 24: Contributions from the bottom QW states in ARPES images. Comparison of (**a**) 6-ML $SrVO_3$ QW structures with (**b**) 4-ML $SrTiO_3$/ 6-ML $SrVO_3$ QW ones. These ARPES images are normalized to the incident photon flux. It is hard to see the signal from the buried metallic QW states for 4-ML $SrTiO_3$/ 6-ML $SrVO_3$ QW structures in this intensity scale. **c**, Comparison of ARPES spectra at the Γ point.

Supplementary Note 13. Quantum confinement at vacuum/$SrVO_3$ and $SrTiO_3$/$SrVO_3$ interfaces

Supplementary Figure 25 presents a comparison of ARPES images between vacuum/ 6-ML $SrVO_3$ QW structures and 4-ML $SrTiO_3$/ 6-ML $SrVO_3$ (T_4V_6) QW structures (the same as in Supplementary Fig. 24, but with the dynamic range expanded by a factor of 20 for Supplementary Fig. 25b). When the dynamic range in the T_4V_6 image is expanded, we observed very weak but distinct QW states from the bottom QW. Because $SrTiO_3$ is an *n*-type semiconductor with a bandgap of 3.2 eV, the $SrTiO_3$ overlayer does not mask the QW states derived from the V 3*d* states near E_F of the buried $SrVO_3$ QW structure. Thus, we address the buried $SrVO_3$ QW states (confinement in two $SrTiO_3$ barriers) using ARPES. To evaluate the quantization levels, we show the ARPES spectra at the Γ points in Supplementary Fig. 25c. As can be seen in Supplementary Fig. 25c, it is evident that there is little change in the quantization levels below 200 meV (n = 1 and 2) for both the QW structures. The results demonstrate that the vacuum and $SrTiO_3$ have almost identical contributions to the confinement in the $SrVO_3$ layer[4]. It should be noted that the invariance of the QW states at the surface (vacuum/$SrVO_3$ interface) and the interface ($SrTiO_3$/$SrVO_3$) provide further evidence for the formation of a chemically abrupt interface between $SrTiO_3$ and $SrVO_3$.

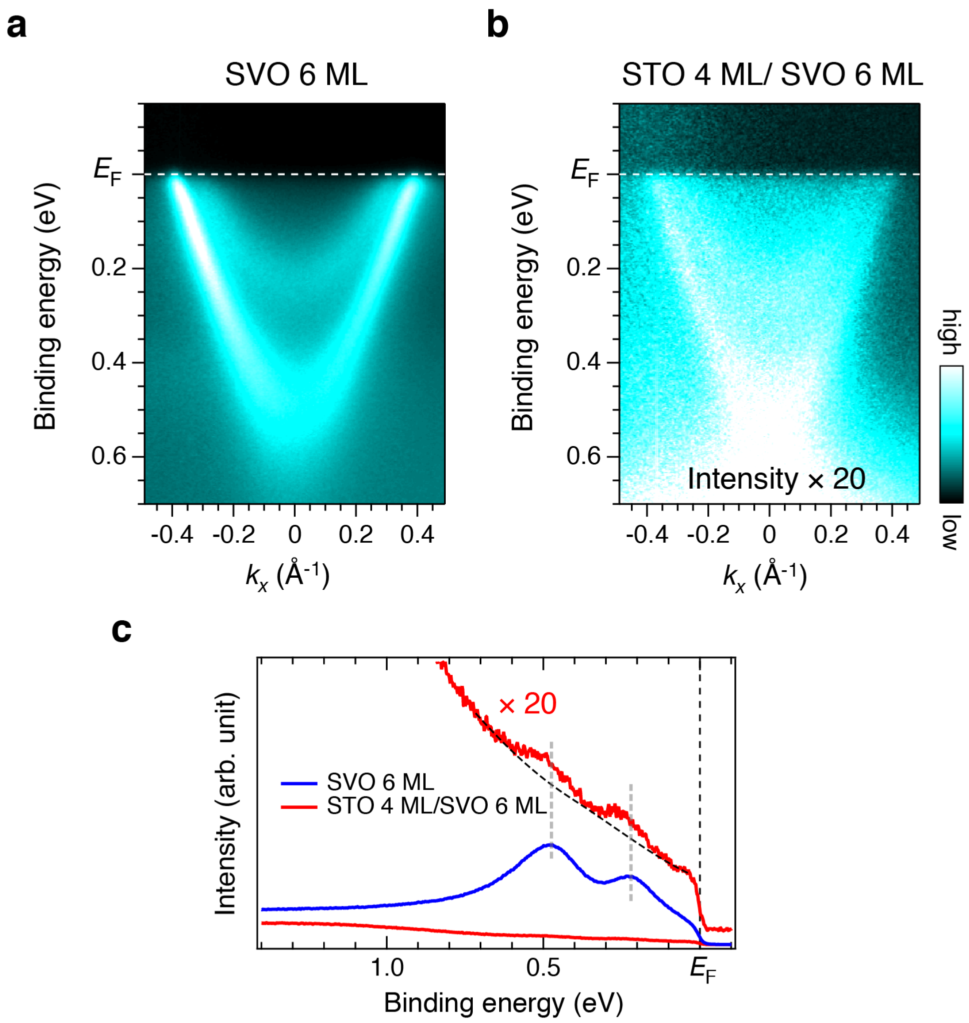

Supplementary Figure 25: Quantum confinement at vacuum/$SrVO_3$ and $SrTiO_3$/$SrVO_3$ interfaces. Comparison of ARPES images between (**a**) vacuum/ 6-ML $SrVO_3$ QW structures and (**b**) 4-ML $SrTiO_3$/ 6-ML $SrVO_3$ (the same as Supplementary Fig. 24b, but with the dynamic range expanded by a factor of 20). **c**, ARPES spectra at the Γ points, where the ARPES peak positions correspond to the quantization energies.

Supplementary Note 14. Details of band-structure calculations

Supplementary Figure 26 shows the results of band structure calculations based on density functional theory (DFT) for the double QW structure (left half side) in comparison with the ARPES images taken with LH polarization (right half side). In the band structure calculation, the results for all d_{xy}, d_{yz}, and d_{zx}-derived subbands are presented. Note that the d_{zx}-derived subbands, as well as the ARPES images, are exactly same as those in Fig. 3 in the main text. As described in Supplementary Note 7, d_{zx}-derived subbands are dominant in the ARPES images owing to the dipole selection rules for the present experimental setup. In fact, the flat d_{yz}-derived subbands that degenerate with the d_{zx}-derived ones at the Γ point (k_x = 0 Å^{-1} in Supplementary Fig. 26) are not detected. Thus, the comparison between the calculation and experiment highlights the validity of comparing only d_{zx}-derived subbands with the present ARPES results, as presented in Fig. 3 in the main text.

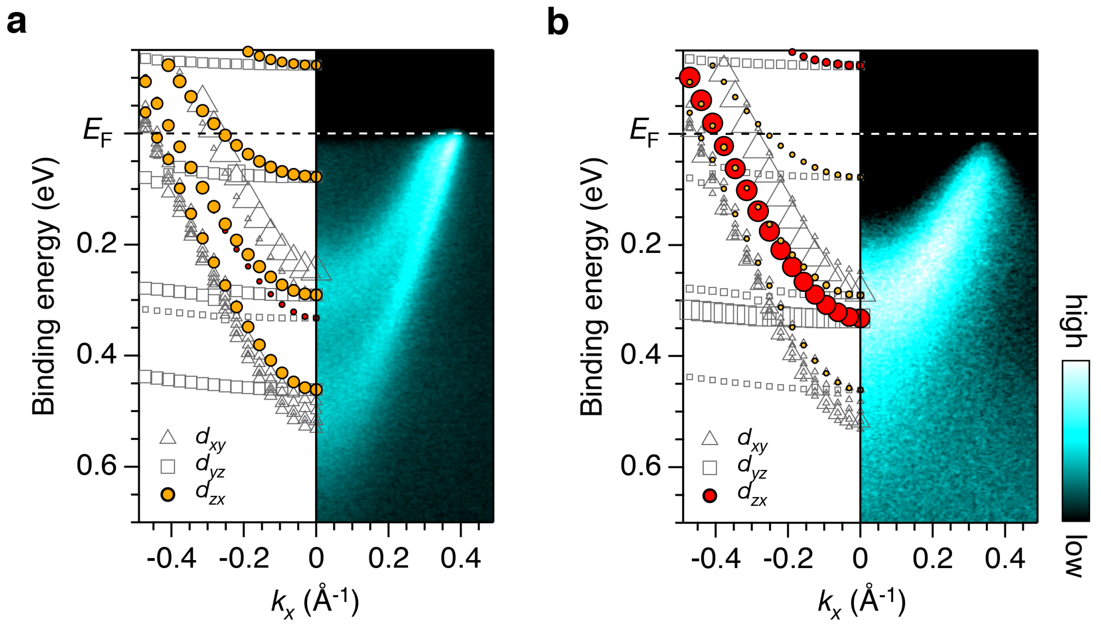

Supplementary Figure 26: DFT calculations for all t_{2g} subbands in double QW structures. Comparison of ARPES images taken along the Γ-X direction with the DFT calculations for $V_6T_2V_2$ (**a**) and $V_2T_2V_6$ (**b**) double QW structures. Raw ARPES intensity plots are shown on the right half side and the DFT calculations on the left half side. Triangles, squares, and circles represent the d_{xy}-, d_{yz}-, and d_{zx}-derived bands, respectively. The probability of the electron being

detected by ARPES is represented by the size of the markers. Since only d_{zx}-derived subbands are detected in the present experimental configuration (Supplementary Fig. 12), the corresponding subbands with a predominant d_{zx} character are highlighted in the figures as filled circles for comparison. Note that the d_{zx}-derived subbands and the ARPES images are exactly same as those in Fig. 3 in the main text.

Supplementary Note 15. DFT calculations for subband structure formed in the $V_2T_LV_6$ heterostructures

15.1 Barrier-layer-thickness dependence

Supplementary Figure 27 shows the DFT results for subband structures formed at the $V_2T_LV_6$ heterostructures to demonstrate how the resonant tunneling (RT) changes as a function of barrier-layer thickness L. Systematic evolution of the RT effects between the two QW states is observed with reducing L. The DFT calculations show the formation of four d_{zx}-derived subbands (n' = 1–4) from the bottom. For L = 1, because of the significant hybridization between the original n = 1 of the 2-ML $SrVO_3$ QW and n = 2 in the 6-ML $SrVO_3$ QW, these two energetically close quantization levels form bonding (n' = 2) and antibonding (n' = 3) states in the double QW. In contrast, the original n = 1 and 3 states in the bottom 6-ML QW (n' = 1 and 4 states in the $V_2T_LV_6$ double QW structure) are not hybridized to any levels in the top QW and remain unchanged. With increasing L, the hybridization weakens steeply and seems to almost disappear at L = 4. Consequently, in the $V_2T_LV_6$ double QW structures with $L \geq 4$, the top 2-ML QW and bottom 6-ML QW behave as independent QWs.

The evolution of the RT effects is further confirmed by the existence probabilities of electrons belonging to each subband, as shown in the middle panels of Supplementary Fig. 27. To evaluate the RT effect, we calculate the existence probability of n' = 2 states (original n = 1 states of the 2-ML QW) that spreads over the 6-ML QW side ($\rho_{\mathrm{SVO\,6ML}}^{n'=2}$), and plot it in Supplementary Fig. 28 as a function of L. In this plot, the steep attenuation of $\rho_{\mathrm{SVO\,6ML}}^{n'=2}$ with increasing L is observed, reflecting the nature of the tunneling effect. By fitting the data with an exponential decay function of $\exp(-La/\lambda)$ where a is the lattice constant of $SrTiO_3$, we determined the characteristic length scale of the barrier layer required for RT to be λ = 0.33 nm (~0.85 ML). This value is in line with the experimental results shown in Supplementary Fig. 20.

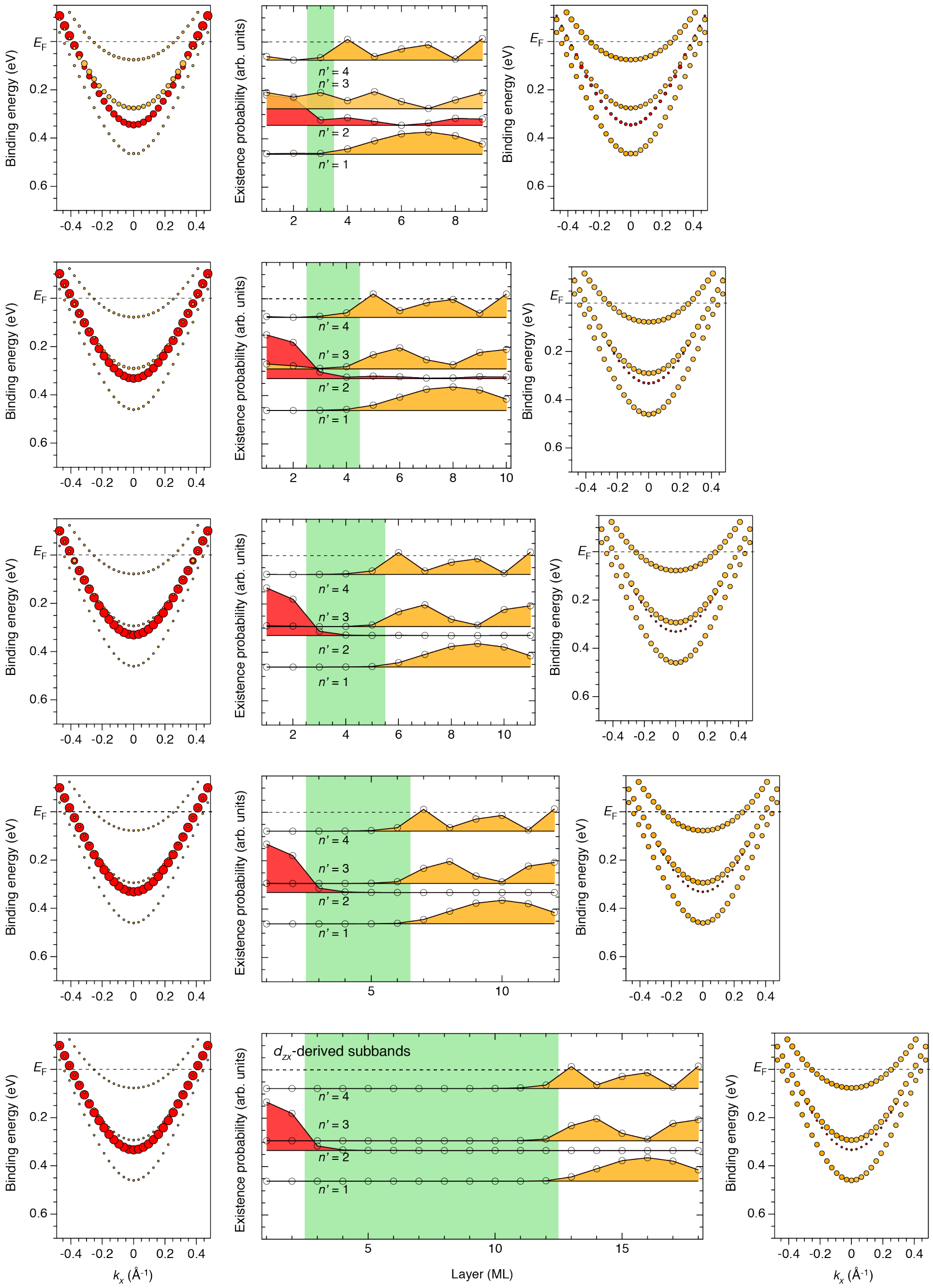

Supplementary Figure 27: DFT calculations for barrier-layer-thickness dependence. DFT results for d_{zx}-derived subband structures formed at the $V_2T_LV_6$ heterostructures (L = 1, 2, 3, 4, and 10 from top). The results of DFT calculation for the top 2-ML (bottom 6-ML) QWs are shown on the left- (right-) hand panels, where the probability of the electron being detected by ARPES is presented by the size of the filled circles. Existence probabilities for the respective d_{zx}-derived subbands, which are calculated by Mulliken population analysis, are plotted along the z direction for the $V_2T_LV_6$ heterostructures. The baselines of the existence probabilities (solid

black lines) correspond to the quantization energies (subband minimum energies). Note that the DFT results are presented in the same manner as in Fig. 3 in the main text, while the result for $V_2T_LV_6$ with $L = 2$ is the same as that in Fig. 3.

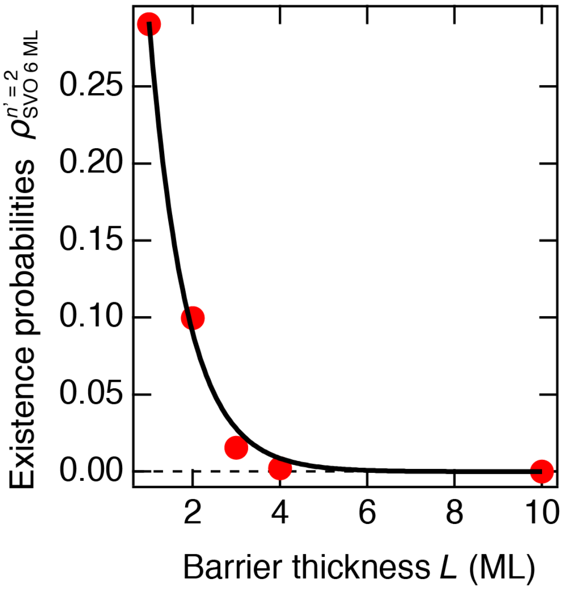

Supplementary Figure 28: Calculated existence probability in the $V_2T_LV_6$ heterostructures. The plot of the existence probabilities of n' = 2 states in the 6-ML QW side ($\rho^{n'=2}_{SVO\ 6ML}$) as a function of L. The data points are fitted to an exponential decay function (a solid line).

15.2 Termination-layer dependence

Supplementary Figure 29 shows the DFT calculations for VO_2-terminated (the same as those in Fig. 3 in the main text) and SrO-terminated $V_2T_2V_6$ structures. At first glance, there is no significant difference between the two. However, a closer look reveals that the QW states in the SrO-terminated 2-ML $SrVO_3$ QW (n_{2ML}) rigidly shift toward lower binding energies, resulting in a resonant tunneling (RT) effect between $n_{2ML} = 1$ and $n_{6ML} = 3$ ($n_{2ML} = 2$ and $n_{6ML} = 5$), owing to the proximity of the respective quantization energy levels. Although the weak but distinct hybridization effect in the SrO termination is also confirmed by the existence probability shown in Supplementary Fig. 30, the RT effect in the SrO termination is much weaker than that in the VO_2 termination, reflecting the larger difference in the quantization energies of the original QW states.

It should be noted that the termination layer of the fabricated double QW structure is a VO_2 atomic layer, since we used TiO_2-terminated $SrTiO_3$ substrates[19]. Thus, we compared the ARPES results with the DFT calculations for the VO_2-terminated $V_2T_2V_6$ structure. Indeed, as can be seen in Fig. 3 in the main text, the experimental data show much better agreement with the VO_2-terminated $V_2T_2V_6$ structure.

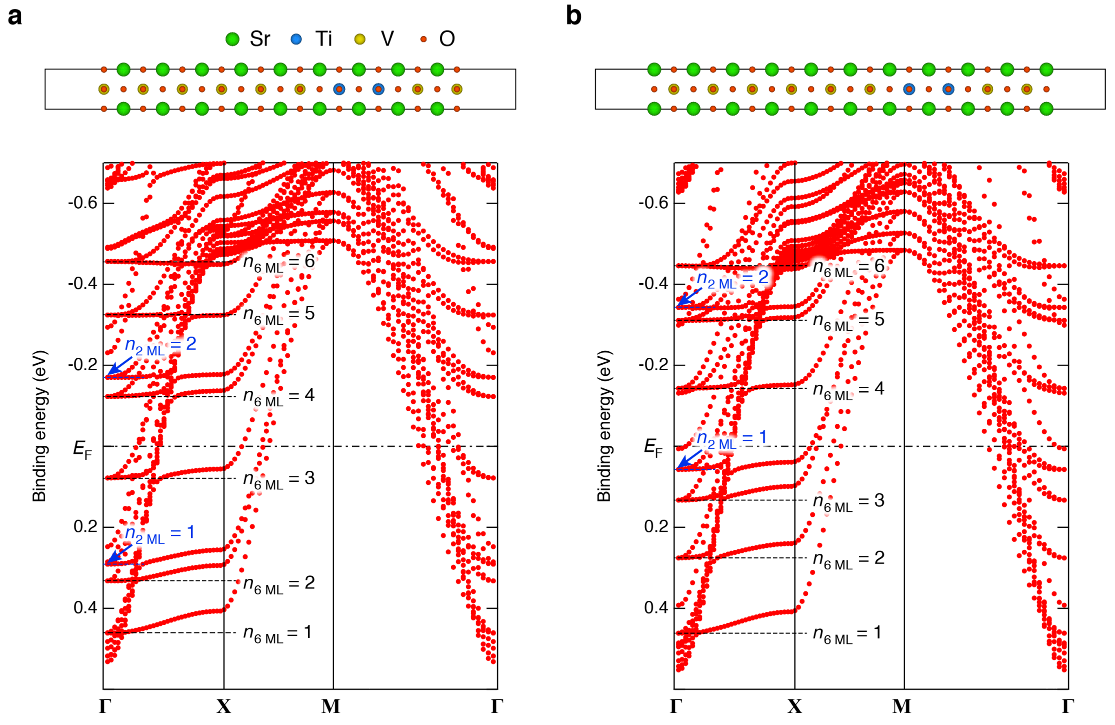

Supplementary Figure 29: DFT calculations for termination-layer dependence. DFT calculation results for (**a**) VO_2-terminated $V_2T_2V_6$ (Vac./ VO_2-SrO-VO_2-SrO-TiO_2-SrO-TiO_2-SrO-VO_2-SrO-VO_2-SrO-VO_2-SrO-VO_2-SrO-VO_2-SrO-VO_2/ Vac. slab) and (**b**) SrO-terminated $V_2T_2V_6$ (Vac./ SrO-VO_2-SrO-VO_2-SrO-TiO_2-SrO-TiO_2-SrO-VO_2-SrO-VO_2-SrO-VO_2-SrO-VO_2-SrO-VO_2-SrO-VO_2-SrO/ Vac. slab) double QW structures. The notations indicate the corresponding quantum number (n) of the original QW structures. The structure models are shown in the top panels.

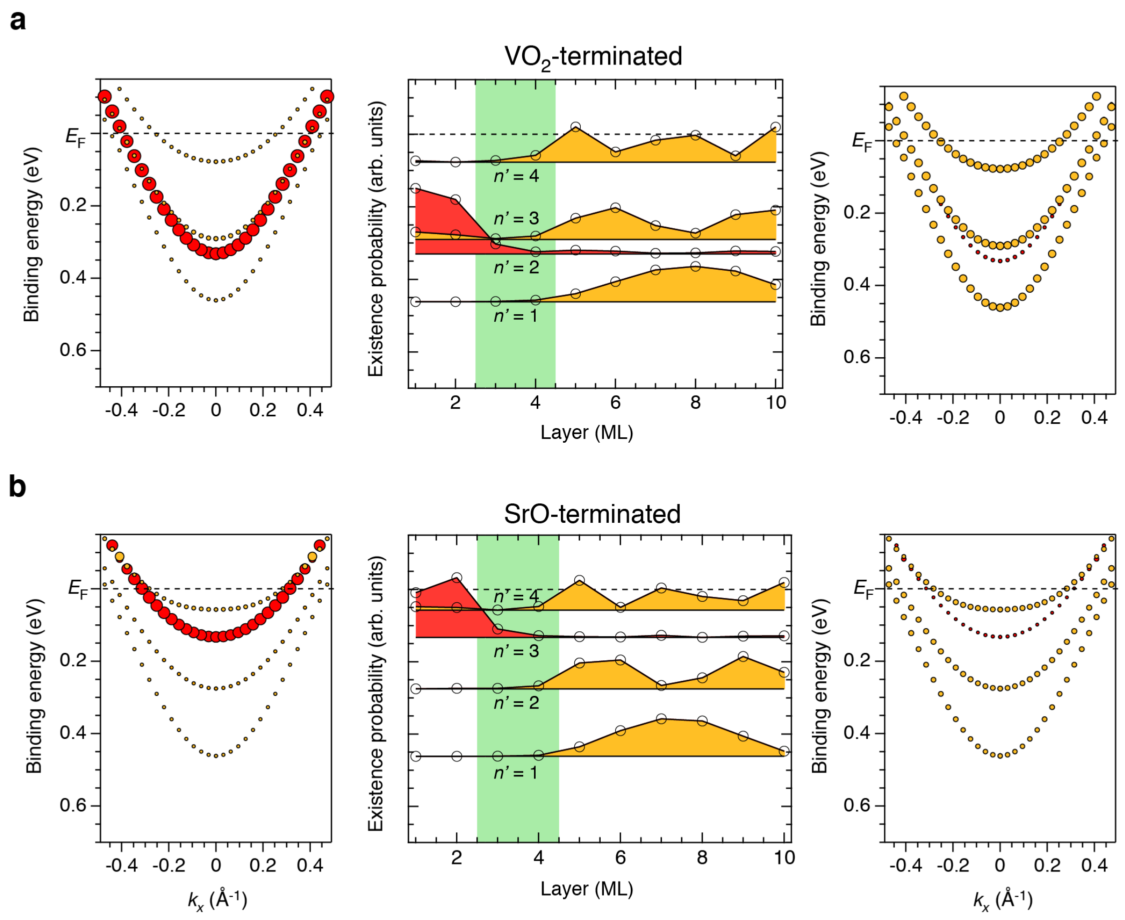

Supplementary Figure 30: Subband structures formed at the termination-dependent $V_2T_2V_6$ heterostructures. DFT results for d_{zx}-derived subband structures formed at the VO_2-terminated (**a**) and SrO-terminated (**b**) $V_2T_2V_6$ heterostructures. The results of DFT calculation for the top 2-ML (bottom 6-ML) $SrVO_3$ QWs are shown on the left- (right-) hand panels, where the probability of the electron being detected by ARPES is presented by the size of the filled circles. Existence probabilities for the respective d_{zx}-derived subbands, which are calculated by Mulliken population analysis, are plotted along the z direction for the $V_2T_2V_6$ heterostructures. The baselines of the existence probabilities (solid black lines) correspond to the quantization energies (subband minimum energies). The upward shift of $n' = 3$ for SrO-terminated $V_2T_2V_6$ may arise from the charge transfer from a top 2-ML QW to the topmost SrO layer and the resultant reduction of the population (chemical potential shift) in the original 2-ML QW structures.

Supplementary References

1. Sze, S. M. & Ng, K. K. *Physics of Semiconductor Devices* 3rd edn (Wiley–Interscience, 2006).

2. Hubbard, J. Electron Correlations in Narrow Energy Bands. *Proc. R. Soc. A Math. Phys. Eng. Sci.* **276**, 238–257 (1963).

3. Yoshimatsu, K. *et al.* Metallic Quantum Well States in Artificial Structures of Strongly Correlated Oxide. *Science* **333**, 319–322 (2011).

4. Yoshimatsu, K. *et al.* Determination of the surface and interface phase shifts in metallic quantum well structures of perovskite oxides. *Phys. Rev. B* **88**, 115308 (2013).

5. Kobayashi, M. *et al.* Origin of the Anomalous Mass Renormalization in Metallic Quantum Well States of Strongly Correlated Oxide $SrVO_3$. *Phys. Rev. Lett.* **115**, 076801 (2015).

6. Kobayashi, M. *et al.* Emergence of Quantum Critical Behavior in Metallic Quantum-Well States of Strongly Correlated Oxides. *Sci. Rep.* **7**, 16621 (2017).

7. Zhong, Z., Zhang, Q. & Held, K. Quantum confinement in perovskite oxide heterostructures: Tight binding instead of a nearly free electron picture. *Phys. Rev. B* **88**, 125401 (2013).

8. Yoshimatsu, K. *et al.* Dimensional-Crossover-Driven Metal-Insulator Transition in $SrVO_3$ Ultrathin Films. *Phys. Rev. Lett.* **104**, 147601 (2010).

9. Minohara, M., Yasuhara, R., Kumigashira, H. & Oshima, M. Termination layer dependence of Schottky barrier height for $La_{0.6}Sr_{0.4}MnO_3$/Nb:$SrTiO_3$ heterojunctions. *Phys. Rev. B* **81**, 235322 (2010).

10. Shiga, D. *et al.* Thickness dependence of electronic structures VO_2 ultrathin films: Suppression of the cooperative Mott-Peierls transition. *Phys. Rev. B* **102**, 115114 (2020).

11. Mossanek, R. J. O. *et al.* Evolution of the spectral weight in the Mott-Hubbard series $SrVO_3$-$CaVO_3$-$LaVO_3$-YVO_3. *Phys. Rev. B* **78**, 075103 (2008).

12. Bourlier, Y. *et al.* XPS monitoring of $SrVO_3$ thin films from demixing to air ageing: The asset of treatment in water. *Appl. Surf. Sci.* **553**, 149536 (2021).

13. Fouchet, A. *et al.* Interface chemical and electronic properties of $LaAlO_3$/$SrVO_3$ heterostructures. *J. Appl. Phys.* **123**, 055302 (2018).

14. Lin, C., Posadas, A., Hadamek, T. & Demkov, A. A. Final-state effect on x-ray photoelectron spectrum of nominally d^1 and *n*-doped d^0 transition-metal oxides. *Phys. Rev. B* **92**, 035110 (2015).

15. Koitzsch, A. *et al.* In-gap electronic structure of $LaAlO_3$-$SrTiO_3$ heterointerfaces investigated by soft x-ray spectroscopy. *Phys. Rev. B* **84**, 245121 (2011).

16. Kumigashira, H. *et al.* *In situ* photoemission characterization of terminating-layer-controlled

$La_{0.6}Sr_{0.4}MnO_3$ thin films. *Appl. Phys. Lett.* **82**, 3430 (2003).

17. Kobayashi, D. *et al.* High-resolution synchrotron-radiation photoemission characterization for atomically-controlled $SrTiO_3$(001) substrate surfaces subjected to various surface treatments. *J. Appl. Phys.* **96**, 7183 (2004).

18. Zhang, P. *et al.* A precise method for visualizing dispersive features in image plots. *Rev. Sci. Instrum.* **82**, 043712 (2011).

19. Okada, Y. *et al.* Quasiparticle Interference on Cubic Perovskite Oxide Surfaces. *Phys. Rev. Lett.* **119**, 086801 (2017).
